# Agents of change: Comparing HIV-related risk behavior of people attending ART clinics in Dar es Salaam with members of their social networks

**DOI:** 10.1371/journal.pone.0238240

**Published:** 2020-09-04

**Authors:** Sylvia Kaaya, Hellen Siril, Keith McAdam, Donald Ainebyona, Magreat Somba, Elspeth McAdam, Kicki Oljemark, James Todd, Irene Andrew, Alice Simwinga, Neema Mleli, Samwel Makongwa, Yuanyuan Liu, Jeffrey Lienert, Sabina Haberlen, Mary C. Smith Fawzi

**Affiliations:** 1 Department of Psychiatry and Mental Health, Muhimbili University of Health and Allied Sciences, Dar es Salaam, Tanzania; 2 Management and Development for Health, Dar es Salaam, Tanzania; 3 Namweza Center, United Kingdom and Sweden; 4 Department of Population Health, London School of Hygiene and Tropical Medicine, Mwanza, Tanzania; 5 Department of Health, City of Dar es Salaam, Dar es Salaam, Tanzania; 6 Department of Global Health and Social Medicine, Harvard Medical School, Boston, MA, United States of America; 7 Department of Epidemiology, Johns Hopkins Bloomberg School of Public Health, Baltimore, MD, United States of America; Pontificia Universidade Catolica do Rio Grande do Sul, BRAZIL

## Abstract

The aim of the study is to compare sociodemographic characteristics, psychosocial factors, HIV knowledge and risk behaviors of people living with HIV (PLH) and their social network members (NMs) to inform HIV prevention programs that engage PLH as prevention educators in their communities. We compared baseline characteristics of PLH enrolled in an intervention to become HIV prevention Change Agents (CAs) (n = 458) and 602 NMs they recruited. CAs and NMs responded to questionnaires through a computer-driven interface with Audio Computer-Assisted Self Interview (ACASI) software. Although NMs scored higher on socio-economic status, self-esteem and general self-efficacy, they had lower HIV knowledge (AOR 1.5; 95% CI: 1.1–2.1), greater inconsistent condom use (AOR 3.2; 95% CI: 2.4–4.9), and recent experience as perpetrators of physical (AOR 2.5; 95% CI: 1.2–5.1) or sexual (AOR 4.1; 95% CI: 1.4–12.7) intimate partner violence; and as victims of physical (AOR 1.5; 95% CI: 1.0–2.3) or sexual (AOR 2.2; 95% CI: 1.3–3.8) forms of violence than CAs. Higher HIV knowledge and lower sexual risk behaviors among CAs suggest PLH’s potential as communicators of HIV prevention information to NMs. CAs’ training should also focus on improving self-esteem, general self-efficacy and social support to increase their potential effectiveness as HIV prevention educators and enhance their own overall health and well-being.

## Introduction

Despite a 42% decline in AIDS-related mortality and a 30% decline in new HIV infections in the sub-Saharan African region between 2010–2017, 59% of the estimated 980,000 million global new infections in 2017 occurred amongst women in the region [1]. While HIV prevalence rates in Tanzania have declined from an estimate of 5.1% in 2012 to 4.7% in 2016, the incidence rate of 0.29% reflects 81,000 new annual HIV infections in this period [2, 3]. Scale up of ART access in HIV services in Tanzania, has improved survival in PLH [4, 5], transforming HIV/AIDS into a manageable chronic infectious disease. However, concerns of health workers’ abilities to meet the information needs of PLH attending high volume clinics exist [6]. The chronic nature of HIV/AIDS, within an overburdened health delivery system may pose challenges to sustaining adherence and implementing clinic-based interventions aimed at minimizing HIV transmission risk behaviors in PLH.

Most studies among PLH receiving ART in sub-Saharan Africa (SSA) do not find associations between ART initiation and increased HIV transmission risk behaviors. Recent reviews of studies assessing sexual behavior outcomes among PLH receiving ART in SSA show that most engage in HIV risk reduction behaviors when receiving related messages within ART services [7] or specifically tailored prevention interventions [8]. Effects on sexual risk behaviors for HIV prevention interventions included 57% higher HIV status disclosure rate to sexual partners, higher sexual abstinence and condom use rates at last sexual intercourse, and reductions in numbers of sexual partners among those receiving the intervention [8]. However, evidence for persistent risk behaviors in PLH accessing treatment exist. For example, 24% of PLH accessing care and enrolled in a HIV transmission prevention intervention, reported inconsistent condom use at baseline assessment, suggesting the need to identify and work with PLH with higher transmission risks, by intensifying HIV prevention services [9]. Intimate partner violence is increasingly recognized as a risk factor that increases HIV susceptibility and predicts poor outcomes in PLH, affecting retention in care, ART adherence, mental health and ability to negotiate for safe sex practices [10, 11]. Studies to determine associations between IPV and HIV serostatus, have particularly focused on women in SSA, most showing positive associations between various forms of IPV and HIV [12–17]. There is, however, much less information focusing on IPV in men and associations with HIV in SSA.

Effective HIV transmission prevention strategies include supportive prevention efforts, using both behavioral and biologic strategies. These are administered for and tailored to the needs of PLH, aiming to lower infection risks with other pathogens, and benefiting public health by limiting transmission to non-infected sexual partners [18–20]. Meta-analyses of studies reporting behavioral interventions designed for PLH from high income countries show feasible (72%) participant retention rates [21], and significant reductions in reported unprotected sex [22], and sexually transmitted diseases [21, 22]. Few evaluations of peer-facilitated HIV prevention interventions tailored for PLH are available from SSA, and findings from high income countries may not be transferable to SSA related to differences in cultural and socioeconomic contexts as well as the generalized nature of the HIV epidemic in SSA. However, treatment assistants of PLH are a unique SSA peer support solution. They augment health care workers by improving retention in care and ART adherence [23–25]. Qualitative evidence from three SSA countries shows treatment assistants are selected by PLH from their social networks and engage with them in supportive communications that improve the social health of PLH including reducing social difference and increasing hope, adaptive coping with stigma, and responsiveness to HIV status disclosure [23]. There is also some evidence for trained PLH or peer-navigators having positive effects on improving linkage to and retention in care amongst PLH [26]. There is less information on PLH effectivess as HIV transmission prevention advocates in communities, though a study in Uganda reported on willingness of PLH to engage in ongoing HIV preventive communications in their communities [27]. Given the improved PLH survival and retention in HIV care in urban Tanzania [4, 25], PLH may be a potential resource for HIV transmission prevention messaging. More information from the region is needed to inform their utility as HIV prevention communicators to social networks beyond sexual partners.

The parent study providing data for these analyses implemented and evaluated a HIV prevention intervention (*NAMWEZA*—a Swahili truncation of “yes together we can”) that aimed to empower PLH accessing ART services to be change agents (henceforth referred to as CAs) in their communities [21, 22]. The objective of these analyses is to compare PLH and their social network members, who were recruited by the CAs and were typically family members or close friends, on psychosocial health status, HIV prevention knowledge, IPV, and reported condom use behaviors at baseline. We hypothesize that PLH experiences with services, may result in demonstrable higher levels of HIV prevention knowledge and consistent condom use as well as lower levels of IPV than their NMs. However, we also hypothesized, in part due to HIV related stigma, that PLH may demonstrate poorer psychosocial functioning compared to their NMs. The findings may inform the content of programs that intend to engage PLH as community-based HIV transmission prevention educators.

## Materials and methods

### Study design and population

A cross-sectional analysis is reported of baseline data from a stepped wedge randomized controlled trial of an HIV prevention intervention conducted from February 2012 to January 2014 described elsewhere [25, 28]. PLH volunteers were recruited and enrolled from an HIV care and treatment clinic and its satellite clinics in the Kinondoni Municipality of Dar es Salaam, Tanzania. Social network members of recruited potential CAs, were invited by the PLH to participate in surveys and were also enrolled.

We advertised the intervention, henceforth called the *NAMWEZA* program, through presentations and brochures at the two study clinics, and consented and enrolled PLH volunteering to be trained to become HIV transmission prevention change agents in their communities. Recruited CAs were screened for eligibility and enrolled after meeting additional criteria and providing informed consent. Recruited potential CAs were invited to study orientation meetings where they also shared skills for inviting NMs to surveys; they were enrolled as CAs if they were able to invite two or more NMs for surveys. Inclusion criteria for CAs were: 1) being on antiretroviral therapy (ART) for at least three months; 2) aged 18 years old and above; 3) living in the Kinondoni municipality and planning to live there for at least the next two years; 4) provision of informed consent; 5) willingness to engage in HIV prevention actions post training; and 6) attending a study orientation workshop and inviting at least two social network members (NMs) whom they felt were at risk of HIV or intimate partner violence (IPV) to take part in the study. Clinical assessments excluded CAs who were not well enough to attend the training. CAs gave invited NMs a study card with instructions on how to contact a study staff member for eligibility screening. NMs’ inclusion criteria were: 1) 18 years of age or greater; 2) living in Dar es Salaam region and planning to remain in the area for at least two years; and 3) invited by their CA to take part in the study. Exclusion occurred for NMs invited by another CA and those unable to provide informed consent. NMs in these analyses include those who provided consent and completed the baseline survey. Both CAs and NMs responded to questionnaires during the same timeframe using Audio Computer Assisted Self-Administered Interview (ACASI) software with help when needed from a trained facilitator. Use of ACASI compared to traditional face-to-face interviews increases responsiveness to sensitive questions and decreases social desirability in responses [29, 30]. NM surveys occurred at a separate non-HIV clinic study site, to protect CAs’ confidentiality in the event HIV serostatus disclosure to nominated NMs had not occurred.

### Measures

Both CAs and NMs provided information on their HIV transmission contexts, risk behaviors and sociodemographic measures. We also assessed CAs on psychosocial health status measures, including HIV stigma, attitudes toward HIV prevention, perceived social support and level of depressive symptoms.

#### HIV transmission contexts and risk behaviors

Measures included HIV knowledge, condom use in the past six months, exposure to intimate partner violence, general and specific HIV prevention self-efficacy and self-esteem. Five of the 12 HIV knowledge items were derived from the Tanzania HIV and Malaria Indicator Survey [2] and seven additional items were informed by formative phase findings of relevant HIV transmission myths and knowledge of vertical transmission of HIV. Correct and incorrect responses to knowledge items were scored 1 and 0 respectively. Inadequate HIV prevention knowledge was defined as less than 70% correct on this scale. Information on condom use in the past six months was obtained through items from the Prevention with Positives Study conducted in Tanzania [9] and assessed condom use with all sexual partners using 3-point response options of “never”, “sometimes”, or “at all times”. We defined inconsistent condom use as responses of “never” or “sometimes” using a condom with any sexual partner. A yes/no response format assessed use of alcohol in the past six months. In addition, items from the domestic violence module of the Tanzania Demographic and Health Survey [2] assessed past six months of victimization (9 items) and perpetration (9 items) for sexual and physical forms of IPV, using a “yes” or “no” response format.

#### General and specific self-efficacy

The General Self-Efficacy Scale [31] assessed confidence in coping in a series of stressful situations. It included five items with four-point response options [32]. We assessed specific self-efficacy for condom use, HIV disclosure and HIV prevention advocacy using 13 adapted items from the Ghanaian version of the Condom Use Self Efficacy Scale that has been used in similar settings [33, 34]. Additional items for assessing confidence in communicating HIV disclosure and HIV prevention advocacy messages to partners and peers were added (see Table 1). A four-point response format from “not at all” to “very confident” was used. Seven of 10-items from the Rosenberg Self-Esteem Scale [35] assessed self-esteem using a four-point response format [36]. Higher values corresponded with greater self-efficacy and self-esteem for these scales.

**Table 1:** Summary description of scales, internal reliability and preliminary construct validity analyses.

Measure	Response options	Items	Cronbach’s alpha this study (other studies)	Significant correlations (*r)* in the expected directions in this study
Domestic Violence Module of the Tanzania Demographic and Health Survey and Malaria Indicator Survey [2]	Dichotomous Yes/No	Adapted 18 Items; 9 each for IPV perpetration and victimization (Physical violence 7 items; Sexual violence 2 Items)	*Victimization* Physical: 0.86; Sexual: 0.79 *Perpetration* Physical: 0.75; Sexual: 0.52	
General Self-Efficacy Scale (GSES) [22–23]	4-point scale; more general self-efficacy with higher scores	5 items assessing confidence in coping in a series of stressful situations. Sample items include; if someone opposes me, I can find the means and ways to get what I want and thanks to my resourcefulness, I know how to handle unforeseen situations.	0.76 (0.76–0.9)	Self-esteem (r *=* 0.27)
Specific self-efficacy for safe sex, disclosure and HIV prevention advocacy [24–25]	4-point scale; more specific self-efficacies with higher scores	13 items; four for condom use self-efficacy: Confident that; you know where to get condoms wherever you are, you can make sure you have condoms with you all the time, you can use a condom correctly and consistently when you have had a drink, you can refuse to have sex with your partner if he/she refuses to use a condom; and additional scale items for safe sex and HIV prevention. communications including confidence in communicating about the importance of correct and consistent condom use with a) sexual partners and b) family and friends; confidence in communicating with family and friends about c) how to protect themselves against HIV infection, d) the importance of getting tested for HIV e) the importance of accessing HIV care and treatment and f) the importance of disclosing to others about HIV status.	0.86 (0.79–0.90)	Condom use (r = 0.73)
Self-esteem [26–27]	4-point scale; higher self-esteem with higher scores	7 items; Two (51.4% variance explained) of three constructs locally relevant; a 4 item self-enhancing and a 3 item self-derogation construct	10 item scale: (α = 0.81 to 0.88)	
			7 item scale: α = 0.64	
			Constructs included in these analyses were a self-enhancing: α = 0.74; and Self-derogation construct: α = 0.66	
Perceived Social Support [28–29]	4-point scale; more perceived social support with lower scores	10 items, including instrumental and emotional support	α = 0.90 (α = 0.9)	
Depression—Patient Health Questionnaire (PHQ-9) [30–32]	4-point scale (0–3); more symptom severity with higher scores	9 items, including items on depressed mood, somatic symptoms, and self-harm	α = 0.80 (α = 0.78)	
HIV Stigma Scale [33–35]	5-point scale; less HIV related self-stigma with lower scores	7 item scale; 2-item internalized stigma sub-scale (shame, guilt) and 5-item externalized stigma sub-scale	Overall scale: α = 0.64 (α = 0.73 to 0.76)	Self-esteem r = 0.33; GSES r = 0.13; specific self-efficacy r = 0.32; social support r = -0.32; Depression r = -0.16.
			Reliability 2-item subscale in these analyses α = 0.71	
ART-related Attitudes and Beliefs Scale [36]	5-point scale; more positive attitudes with higher scores	9 adapted items measured beliefs and perceptions regarding the effect of ARTs on transmission and prevention of HIV/AIDS with sample items including; I am less concerned about infecting someone because of the availability of antiretroviral drugs (ARVs), Because antiretroviral drugs (ARVs) are becoming increasingly available, I think there is no longer a need for reducing the number of partners.	α = 0.65 (α = 0.61 to 0.66)	

Measures only administered to CAs, included social support, depression and HIV related stigma and ART use and HIV transmission attitudes. Ten of 12-items from the Perceived Social Support Scale [37] which has been used previously in the study context [38] measured functional elements of social support among PLH. The Patient Health Questionnaire (PHQ-9) [39], assessed depressive symptoms and has been used in the region [40–42]. We used a 4-point severity response format to determine if symptoms experienced in the past two weeks affected participants “not at all” to “daily”. Seven items from the HIV Stigma Scale [43] measured HIV-related self-stigma using a 5-point response format ranging from “disagree” to “agree”. The scale has been used in different cultural contexts, including Tanzania [44, 45]. Attitudes were assessed using a 9-item version of the 11-item ART-related Attitudes and Beliefs Scale modified for SSA [46]. The 5-point response options were similar to those of the stigma scale with higher scores reflecting more positive attitudes.

*Sociodemographic measures* that were potential confounders included sex, age, marital status (married or cohabiting, and single or widowed), and proxy socio-economic status measures including; employment status (self-employed, employed or not) and educational attainment (primary, secondary, and post-secondary vocational, and degree level training), assessment of household food security utilizing a single item (if in last 12 months household members had not eaten for the whole day because there was not enough food) with a 5-point response format from “never” to “always”, and presence or not of a private toilet/latrine in the home.

Measures were translated to Swahili and back-translated to English. A panel of bilingual professionals reviewed discrepancies in the English versions, to reach a consensus for final Swahili versions. Piloting measures confirmed meaningful translation of items and capture of local nuances used to describe experiences. Table 1 provides psychometric properties for the scale measures.

### Statistical analysis

To compare baseline sociodemographic characteristics, we calculated frequencies for CAs and NMs for each variable, as well as the odds ratios (ORs) and 95% confidence intervals. For variables that were collected only among the CAs or the NMs, we presented frequencies and corresponding percentages. For bivariate analysis comparing CAs with NMs for the hypothesized outcomes, we either calculated frequencies and chi-square statistics for categorical variables (HIV prevention knowledge, inconsistent condom use, IPV, and alcohol use) or means, standard deviations, and t-tests for continuous variables (general self-efficacy, self-esteem, and self-efficacy for safe sex). We used logistic regression to compare NMs with CAs for HIV prevention knowledge, psychosocial health status variables, and IPV. Multivariable regression models were adjusted for age, sex, employment status, food insecurity, and latrine status, in order to control for any confounding effects of sociodemographic and economic factors for the two groups. Examination of interaction terms of NM/CA status and other covariates in multivariable models occurred. All analyses used the statistical software package (SAS), Release 9.2 (Cary, NC).

### Ethical considerations

The study was approved by the Institutional Review Boards (IRBs) at the Harvard School of Public Health (HSPH), Muhimbili University of Health and Allied Sciences (MUHAS), and the National Institute for Medical Research (NIMR) in Tanzania. A written informed consent was obtained from all study participants.

## Results

Of 753 potential CAs approached, 652 (86.6%) met initial inclusion criteria and provided informed consent. Only 458 (70.2%) met the final inclusion criteria, by inviting two NMs to take part in surveys. Among 690 NMs that study staff could reach by phone, 602 (87.2%) were eligible, provided consent, and completed baseline surveys. Table 2 summarizes sociodemographic and proxy economic status measures for 458 CAs and 602 NMs. Almost 60% of study participants were female and NMs were almost five times more likely to be aged 18–30 years compared to CAs. NMs compared with CAs varied significantly on proxy socio-economic variables.

**Table 2:** Baseline socio-demographic, economic and relationship characteristics of clinic-based change agents and community-based network members in Dar es Salaam, Tanzania.

Characteristic	Network Members N = 602 n (%)	Change Agents N = 458 n (%)	Odds Ratio (95% CI)
Socio-demographic and economic			
Female	327 (54.5)	261 (57.0)	0.90 (0.71–1.16)
18–30 years old	291 (48.0)	75 (16.4)	4.78 (3.56–6.42)
Married or cohabiting	316 (52.7)	234 (51.7)	1.04 (0.82–1.33)
Incomplete primary education	26 (4.0)	30 (7.0)	0.65 (0.38–1.11)
Unemployed	183 (31.0)	208 (45.0)	0.53 (0.41–0.68)
Often or always not satisfying food needs	60 (10.0)	89 (19.0)	0.46 (0.32–0.66)
Lack of a private toilet	233 (39.0)	209 (46.0)	0.76 (0.59–0.97)
Network members reported relationship to change agents			
Friend	258 (43.0)		
Family Member	249 (41.5)		
Support group member	43 (7.2)		
Sex/relationship partner	20 (3.3)		
Church Member	14 (2.3)		
Other	16 (2.7)		
Network members self-reported HIV status			
Positive	54 (9.0)		
Negative	363 (60.3)		
Missing response for HIV test result	27 (4.5)		
Never tested for HIV	158 (26.2		

NMs were 54%, 47%, and 24% less likely than CAs to report low household food security, unemployment, and absence of a private toilet in the home, respectively. However, NMs and CAs were similar in levels of educational attainment. A large proportion of NMs reported their nominating CAs were friends (43%) or family members (42%). About 73.8% of NMs had even tested for HIV and 60% reported their test results to be negative (See Table 2).

Table 3 summarizes CAs responses to psychosocial and HIV prevention socio-cognitive measures. Most CAs, showed predominantly favorable attitudes towards HIV transmission prevention. For example, only 9.0% -19% agreed that using ART reduced concerns about condom use or infecting others. However, a quarter and over a half, agreed that because they were using ART, it was not necessary for them to reduce their sexual partners or disclose HIV status to sexual partners respectively. Internalized HIV stigma is suggested by CA’s high endorsements of guilt (42%) and shame (45%) for being HIV positive; 15% and 17% reported insufficient financial and transportation support respectively. Half of the change agents endorsed a depressed mood, with rates not varying significantly by sex. Almost two thirds (61%; n = 127) of CAs expressing depressed mood reported consequent functional difficulties that were reported more by men than women (66.0% versus 56.8% respectively; (p = 0.046).

**Table 3:** Descriptive characteristics of attitudes towards HIV prevention and psychosocial health measures for Change Agents in Dar es Salaam, Tanzania (n = 458).

Characteristics		Total n (%)
***Attitudes about ART and Transmission Prevention***		
Less concerned about infecting someone due to ART	Agree	41 (9.0)
No need to reduce the number of partners	Agree	113 (24.7)
No need to use condoms	Agree	87 (19.0)
No need to disclosure to sexual partners	Agree	269 (58.7)
***HIV stigma and discrimination***		
Feel guilty about being HIV positive	Yes	194 (42.4)
Feel ashamed about being HIV positive	Yes	204 (44.5)
***Social support (% positive responses)***		
Get visits from friends and relatives	As much as I would like	264 (57.6)
Get help with money in emergency	As much as I would like	70 (15.3)
Get help with transportation	As much as I would like	78 (17.0)
Get love and affection	As much as I would like	184 (40.2)
***Depression (% negative responses)***		
Feeling down, depressed or hopeless	Yes	212 (46.3)
Any difficulty in carrying out daily activity, due to depressive symptoms	Yes	277 (60.7)

CAs were compared to their NMs on HIV transmission prevention knowledge and condoms and alcohol use in Table 4. NMs compared to CAs had lower levels of HIV knowledge (17.2% versus 24.3%; p = 0.03), although this was not a very large difference in absolute terms. In addition, NMs demonstrated a higher recent inconsistent condom use in contrast with CAs (57.2% vs. 31.7%; p<0.005). NMs were also significantly more likely to endorse being sexually active in the past year, but did not vary in reported use of alcohol. For psychosocial health status measures NM showed significantly higher self-esteem scores than CAs, with mean scores 3.08 and 2.99 (p = 0.004) respectively. They however were similar in general and specific self-efficacy scores.

**Table 4:** Comparisons of network members (N = 602) and change agents (N = 458) levels of HIV knowledge, recent alcohol and inconsistent condom use, psychosocial health status and IPV in Dar es Salaam, Tanzania.

	Network Members		Change Agents	
	N = 602; n (%)		N = 458; n (%)	
*HIV prevention knowledge*				
Inadequate HIV knowledge		145 (24.1)		79 (17.2) *
Adequate HIV knowledge		456 (75.9)		379 (82.8)
*Safe sex practices*				
Sexually active (past year)				
Yes		414 (68.8**)**		287 (62.7) **^†^
No		158 (26.2)		170 (37.2)
Missing responses		30 (5.0)		1 (0.2)
Condom use in past 6 months				
Consistent		147 (35.5)		179 (62.3) ***
Inconsistent		237 (57.2)		91 (31.7)
Missing responses		30 (7.2)		17 (5.9)
*Alcohol use in past six months*				
Reported		155 (25.7)		143 (31.2)
Denied		431 (71.6)		315 (68.9)
Missing responses		16 (2.7)		-
Psychosocial health status		Mean (SD)		Mean (SD)
Self-esteem (range 1–4)		3.08 (0.45)		2.99 (0.48) **
General Self-efficacy (range 1–4)		3.45 (0.55)		3.38 (0.56)
Self-efficacy for safe sex (range 1–4)		3.34 (0.65)		3.16 (0.64)
*Intimate partner violence*				
Physical abuse perpetration		45 (7.5)		11 (2.4) ***
Physical abuse victimization		96 (16.0)		46 (10.0) **
Sexual abuse perpetration		26 (4.3)		4 (0.9) **^†^
Sexual abuse victimization		66 (11.0)		24 (5.2) **
*Intimate partner violence by sex*	*Males*	*Females*	*Males*	*Females*
Physical abuse perpetration	30 (11.0)	15 (4.6)	8 (4.1) **	3 (1.1) *
Physical abuse victimization	38 (13.9)	58 (17.7)	13 (6.6)	33 (12.6)
Sexual abuse perpetration	17 (6.2)	9 (2.8)	3 (1.5) *	1 (0.4) **
Sexual abuse victimization	24 (8.8)	42 (12.8)	7 (3.6) *	17 (6.5) **

Key

* p<0.05

**p<0.01

***p<0.001

^†^adjusted p-value, Fisher’s exact test, two tailed.

Recent (past 6 months) physical and sexual intimate partner violence was endorsed by both NM and CA populations; with higher rates reported by NMs than CAs, of and of victimization (16% [n = 96 of 504] versus 10% [n = 46 of 412] respectively; p<0.0001), more commonly than perpetration 7.5% [n = 45 of 555] versus 2.4% [n = 11 of 447] respectively; p<0.005). Both forms of sexual and physical abuse victimization were reported in the past 6 months by NMs and CAs. Physical abuse victimization had the highest prevalence in both types of informants (NMs 16% versus CAs 10%; p = 0.005) than sexual violence victimization (NMs 11% versus CAs 5.2%; p = 0.001). NM reported significantly higher rates of physical and sexual abuse victimization than CAs.

NMs and CAs both also reported physical and sexual abuse perpetration, rates being significantly higher again in NMs that CAs (7.5% versus 2.4% (p<0.0001) and 4.3% versus 0.9% (p = 0.001). (See Table 4). Recent IPV experiences desegregated by sex are also summarized in Table 4. Both NM men (11.0%) and women (4.6%) reported significantly higher physical abuse perpetration when compared with their CA counterparts; rates being 4.6% versus 1.1% respectively (p<0.03) in women; and 11.0% versus 4.1% respectively (p<0.005) in men. Similarly, NM men reported significantly higher rates of physical abuse victimization than their CA counterparts (13.9% versus 6.6% respectively; p = 0.01), with insignificant variations in rates reported by NM and CA women (17.7% versus 12.6% respectively; p = 0.11). While men overall had higher rates of reported perpetration of sexual abuse, similar significant variations by NM or CA status observed for physical and sexual forms of abuse victimization occurred for physical (NM men 6.2% versus CA men 1.5%; p = 0.02) and sexual abuse perpetration (NM men 2.8% versus CA men 0.4% respectively; p = 0.03).

We applied multivariable analysis regressing CA/NM status to study outcome variables with CA status as the reference. As summarized in Table 5 we included sex, age, employment, marital status, household food insecurity and private toilet use as potential confounders in all regression models. In the adjusted model the association between being a NM and inconsistent condom use remained significant with more than three times higher odds of inconsistent condom use (OR = 3.2, [95% CI: 2.4, 4.9]) when compared to being a CA (Table 4). Interaction effects were demonstrated with marital status. The effects of being a NM were stronger among married (OR = 4.2, [95% CI: 2.9, 6.2]) than unmarried persons (OR = 2.2, [95% CI: 1.3, 3.6]), suggesting marital status modified the association between CA/NM status and risk of inconsistent condom use.

**Table 5:** HIV prevention knowledge, risk behavior, intimate partner violence, self-esteem and self-efficacy outcomes among Network Members compared to Change Agents (reference group), in Dar es Salaam, Tanzania (n = 458).

Characteristics	Univariate		Multivariable^a^	
	OR (95% CI)	p-value	AOR (95% CI)	p-value
*HIV prevention knowledge and risk behaviors*				
Inadequate HIV knowledge	1.5 (1.1, 2.1)	<0.01	1.5 (1.1, 2.1)	0.02
Inconsistent condom use	3.2 (2.3, 4.4)	<0.001	3.2 (2.4, 4.9)	<0.001
*Intimate partner violence*				
Physical abuse victimization	1.7 (1.2, 2.5)	<0.01	1.5 (1.0, 2.3)	0.05
Physical abuse perpetration	3.3 (1.7, 6.4)	<0.01	2.5 (1.2, 5.1)	0.01
Sexual abuse victimization	2.2 (1.4, 3.6)	<0.01	2.2 (1.3, 3.8)	<0.01
Sexual abuse perpetration	5.1 (1.8, 14.8)	<0.01	4.1 (1.4, 12.7)	0.01
*Self-esteem/ self-efficacy*				
Self-esteem	1.5 (1.1, 1.9)	<0.01	1.4 (1.0, 1.8)	0.04
General Self-efficacy	1.5 (1.3, 1.9)	<0.001	1.5 (1.2, 1.8)	<0.01
Self-efficacy for safe sex	1.2 (0.97, 1.6)	0.09	1.2 (0.90, 1.6)	0.24

^a^ Multivariable model with change agents as reference group, controlling for: age, sex, employment and marital status, food insecurity, and latrine status.

Being a NM compared with being a CA (AOR = 1.5, [95% CI: 1.1, 2.1]) also remained significantly associated with a higher risk of inadequate HIV knowledge, with interaction effects noted for gender. After gender stratification of findings, this association was only significant among NM men who were just over twice more likely (OR = 2.1, [95% CI: 1.3, 3.2]) to demonstrate inadequate HIV knowledge than their CA male counterparts; but this finding was not statistically significant for female NMs when compared to their CA counterparts (OR = 1.1, [95% CI: 0.77, 1.6]).

With respect to intimate partner violence, being a NM remained marginally associated with a higher risk of physical abuse victimization and significantly associated with reporting its perpetration, increasing the odds by 50% (OR = 1.5, [95% CI: 1.0, 2.3]) and two and a half times (OR = 2.5, [95% CI: 1.2, 5.1]) when compared with CAs. Furthermore, being a NM remained significantly associated with reporting sexual abuse victimization and perpetration, increasing the odds over twice (OR = 2.2, [95% CI: 1.3, 3.8]) and four times (OR = 4.1, [95% CI: 1.4, 12.7]), respectively, when compared to being a CA. No interaction term was significant in the model. In the adjusted model as in univariate analyses NMs were 40% and 50% more likely to show higher self-esteem (OR 1.4; [95% CI 1.0, 1.8]). In these models NMs had higher general self-efficacy (OR 1.5; [95% CI 1.2, 1.8]) than CAs, in contrast to univariate analysis findings, while variations in safe sex self-efficacy between NMs and CAs were not significant.

## Discussion

This is one of the few studies comparing HIV transmission risk behaviors and contexts reported by PLH receiving ART services with members of their social networks in SSA. The findings show PLH were not only willing to volunteer as participants, but could invite at least two NMs; providing on average 1.3 NMs for study surveys. CAs compared to NMs, also show higher HIV knowledge, recent consistent condom use, lower intimate partner physical abuse perpetration, and sexual abuse perpetration and victimization. However, NMs were more likely to be employed and with relatively more household assets than CAs, and have higher scores in measures of self-esteem and general self-efficacy.

Exposure to HIV-related information through HIV clinic visits can explain the higher HIV transmission prevention knowledge among CAs. Furthermore, NM men had the lowest level of HIV information, with sex disaggregated analyses showing it was significantly lower than that of their CA counterparts; without this knowledge variance in women NM and CA. This variation may be due to additional knowledge items assessing vertical HIV transmission. NM women compared to men were more likely to have access to such information from antenatal clinics that integrate services for the prevention of maternal to child transmission of HIV.

Attitudes are a known precursor to actual behavior change [47]. Between 75% to 91% of CAs endorsed three of four items assessing HIV prevention attitudes which may heighten their own intentions to undertake safer practices such as consistent condom use and decreasing their risk of IPV perpetration. The 3.2 times higher odds of self-reported inconsistent condom use in NMs compared to CAs may seem counterintuitive, as all CAs were HIV positive, compared to only 9.0% of NMs. The variation may reflect CAs’ greater access to clinic-based information and skills for condom use, as well as distributed condoms. While we did not explore the criteria used by CAs used to select NMs, they may have invited NMs who engaged in less safe sexual behaviors than themselves. The lack of systematic data to understand better this selection process of NM targets, should be an area of future research.

Lower IPV rates in CAs than NMs was an unexpected finding. Similar proportions of CAs and NMs were in cohabiting relationships, however CAs were almost five times more likely to be older than their NMs, and perhaps have more stable marital/cohabiting relationships. In a review of studies to determine IPV risk factors, younger age was a consistent risk factor [48]. This association persisted after controlling for age, suggesting possible effects of a clinic-based implementation of the National Multisectoral Gender-Based Violence (GBV) Prevention Initiative, which was in its third year of implementation during this study [49]. Diffusion at the clinic level, was evidenced by the presence of trained GBV social work or nurse counselor focal persons, who received referrals. We however did not systematically inquire into the utilization by CAs of this service. Finally, IPV survivors may be under-represented in HIV care and treatment settings. A recent study shows only 29% of IPV survivors accessed HIV counseling and testing services, with socio-cultural (shame disclosing GBV and fear of marital discord) barriers identified to access reported [50].

By volunteering to take part in the NAMWEZA intervention, CAs may show an inherent motivation to help others. A recent review of lessons learned from selecting good lay health providers in Africa shows motivation to serve the community was an important selection criterion [51]. From a perspective of diffusion theory, this study may also have captured HIV clinic clients who are early adapters of HIV transmission prevention innovations in communities [52]. CAs may hence be potential agents for diffusing HIV prevention messages amongst those in their high-risk social networks.

These observations boded well for the NAMWEZA intervention for several reasons. First, knowledge and positive attitudes towards preventive actions may increase CAs’ understanding of the underlying principles and rationale of the planned intervention, which will be important for CAs’ skills development for HIV prevention advocacy. Tested clinic-based HIV transmission risk reduction interventions in Africa show moderate effects on transmission prevention with more structured lengthy risk reduction education programs with 1–4 hour sessions held over several weeks [8]. Professional health care providers may not deliver interventions requiring increased patient contact time, in high patient volume HIV clinics in many low-income contexts. Evidence from HIV clinic providers in the study context, for example, shows lower health care provider motivation in high compared to low volume clinics and perception of being less helpful to patients [6]. The planned intervention has the potential to complement HIV risk reduction education by task shifting this responsibility to trained peer lay providers that can potentially add value to existing clinic-based HIV efforts.

Second, the findings support the appropriateness of NMs as CA targets for HIV transmission prevention, as over 60% of NMs reported a HIV negative status. NMs identified by CAs to be at risk of HIV transmission and IPV were friends and family members and likely to be peers of a similar generation, with comparable experiences. Observations show interpersonal communication is more effective in forming and changing attitudes towards issues such as HIV preventive actions; and that subjective evaluations of near-peers who have already adopted change has a greater influence than information based on scientific research provided by experts [52, 53]. Third, there is a possibility for trained CAs to not only engage in HIV prevention advocacy, but to also model HIV risk reduction in their own lives. This may increase their credibility and the adoption of risk reduction strategies in the communities they serve. Implementation by trained peers of HIV prevention interventions increases HIV knowledge and reported condom use with both casual and regular partners amongst heterosexual adult targets [8, 54]. Trained peers may also model healthier intimate relationships as both IPV perpetration and victimization was significantly less prevalent in CAs, and is associated with healthier and safer intimate relationships.

One concern from our findings was CAs and NMs did not differ in scores for HIV prevention self-efficacy defined as an individual’s beliefs in capabilities to execute courses of action to achieve and implement a goal—in this situation HIV transmission prevention. However, findings of high rates of felt stigma, low perceived instrumental and financial support, and depressed mood with related functional impairment could explain the lower self-efficacy. CAs also show lower levels of self-esteem than their network members. While these analyses did not explore associations with serostatus disclosure, the observed high rate of felt stigma and depressive symptomatology, low self-esteem and general self-efficacy may influence PLH’s effectiveness as CAs; that is if a CA does not disclose their HIV status to their NMs, messages concerning HIV prevention may carry less weight. Our observation of poor psychosocial functioning among PLH accessing HIV care and treatment is similar to reports from other SSA contexts [55, 56]. These findings suggest a need to better understand how to overcome psychosocial functioning barriers when developing training strategies to empower PLH to be CAs that deliver HIV transmission prevention communication actions to NMs.

Our findings also show that the NMs of CAs have lower rates of unemployment, better food security and access to essential household needs such as a private toilet. An inherent selection bias when recruiting CAs and NMs may explain the differences in sociodemographic and economic characteristics. CAs volunteers are from public health facilities offering free HIV care and treatment services, targeting persons from lower socio-economic groups in urban Tanzania, with motivation to participate and perhaps, a higher perceived direct utility of the intervention. NMs were persons perceived by CAs to be at high HIV transmission risk and were hence more likely to have greater variations in socio-economic status, and less likely to perceive a direct utility to them of the study.

While younger NMs might enjoy policies expanding access to formal education in Tanzania [57], similar proportions of NMs and CAs had not completed the primary level of the country’s education system. Almost 60% of our CAs are women, and there is evidence that the association between low socio-economic status and HIV transmission risk in urban East Africa may be higher in women [58]. A secondary analysis of the Demographic and Health Survey data showed that adults in Tanzania with six years compared to no formal education were 24% more likely to be HIV infected; and HIV transmission risk correlates positively with wealth [59]. However, it is also likely that CAs were less able to engage in gainful employment due to combinations of chronic illness, felt stigma, and poor psychological health status and because of lower food security and limitations in attaining essential needs for their households.

### Strengths and limitations

A key strength of the study is including data from both PLH and their network member participants in the same analysis, since many studies focus on either PLH (CAs) or the wider population that does not live with HIV (NMs). Furthermore, assessments of IPV explored both male to female and female to male perpetration and victimization. The findings provide insights into approaches to identify and recruit PLH for training as HIV prevention change agents; as well as identifying content areas for focus during training. Using ACASI for data collection may also have generated more accurate information on sexual practices than face-to-face interviews, lowering risk of social desirability in responses.

There are however some limitations to this study. While CAs were requested to invite the NMs they perceived were at risk of HIV acquisition, we did not determine the actual criteria they used to select invited NMs. We were also unable to include all invited NMs in the survey. Some NMs had difficulty texting the study team to express interest—despite providing CAs training that would enable then to assist their NMs. These limitations make it difficult to determine if the observed variations between CAs and NMs is generalizable to other social network members with high transmission risk behaviors or unable to complete expression of interest actions. Since these analyses are cross-sectional, it is not possible to examine the CA/NM relationships over time, which might yield additional insights for HIV prevention interventions. While the use of ACASI for data collection may generate more accurate information on sexual practices than face-to-face interviews, differential reporting bias for HIV risk behaviors may still occur across CAs and NMs. PLH may more likely report more consistent condom use because of social desirability bias associated with their known HIV infection and exposure to the risk reduction messages when receiving HIV care and treatment services.

## Conclusion

Inviting PLH receiving ART to take part in an intervention aiming to train them to become change agents for HIV prevention in their communities resulted in many volunteers, despite fears of inadvertent disclosure of their HIV status. CAs had positive attitudes towards HIV prevention, more prevention knowledge, less recent IPV exposure and lower inconsistent condom use rates than NMs. Furthermore, having less reported recent IPV and more consistent reported condom use suggests PLH potential to influence behaviors of their social networks through modeling safe sex practices. Interventions aiming to train PLH to be CAs should include components that increase self-esteem, comprehensive HIV prevention knowledge, specific self-efficacies for HIV prevention advocacy, and reduce HIV-related self-stigma and symptoms of depression to enhance overall health and well-being in order the maximize their future roles of change agents for HIV transmission prevention in their communities.

## Supporting information

S1 Appendix (PDF)

S2 Appendix (PDF)

S1 File (SAS7BDAT)
