# Supplementary material for: Agents of change: Comparing HIV-related risk behavior of people attending ART clinics in Dar es Salaam with members of their social networks
Source: PLoS One. 2020 Sep 4;15(9):e0238240. doi: 10.1371/journal.pone.0238240 (PMC7473570; doi:10.1371/journal.pone.0238240)
Supplement: S1 Appendix — (PDF) [file pone.0238240.s001.pdf]

Appendix C.1: Interviews with Change Agents: Baseline Questionnaire

CAP MC PHE PROTOCOL

INTERVIEWS WITH CHANGE AGENTS: BASELINE QUESTIONNAIRE

ID number ☐☐☐

PC (Clinic) number ☐☐☐☐☐☐☐☐ ☐☐☐☐☐☐☐

Date of assessment: ☐☐/☐☐/☐☐☐☐ (dd/mm/yyyy)

SECTION 1: SOCIODEMOGRAPHICS			
SD1	WHAT IS YOUR AGE?	Years .... __ __	
SD2	WHAT IS YOUR DATE OF BIRTH?	Day __ __ Month __ __ Year __ __ __ __	
SD3	WHAT IS YOUR GENDER?	Male ....1<br>Female...2	If Male Skip to SD 6
SD4	IF FEMALE, ASK: ARE YOU CURRENTLY PREGNANT?	No .... 2<br>Yes .... 1<br><br>Don't know... 88<br>Refused 77	If No, Don't Know or Refused skip to SD6
SD5	IF YOU ARE CURRENTLY PREGNANT WHEN WAS YOUR LAST MENSTRUAL PERIOD?	Day __ __ Month __ __ Year __ __ __ __	
SD6	WHAT IS YOUR CURRENT MARITAL STATUS?	Married...1<br>Cohabiting ....2<br>Single...3<br>Divorced/Separated ....4<br>Widowed...5<br><br>Refused……………………………………77	
	"I WILL NOW ASK YOU SOME QUESTIONS ABOUT YOUR EDUCATION AND CURRENT EMPLOYMENT."		
SD7	HOW MANY YEARS OF SCHOOL HAVE YOU COMPLETED?<br><br>[COMPLETE ALL CATEGORIES WITH NUMBER OF YEARS OF SCHOOLING; IF YOU HAD NO EDUCATION, INDICATE '0' IN THE TOTAL LINE FOR THIS ITEM.]	Primary Education .... _____<br>Secondary Education .... _____<br>Certificate/Diploma/ Advanced diploma or Other non-degree professional training<br>.. _____<br>University/ Degree level professional training.. _____<br><br>---	
SD8	WHAT IS YOUR CURRENT EMPLOYMENT STATUS?<br><br>[SELECT PRIMARY OCCUPATIONAL STATUS.]	Employed .... 1<br>Self-employed .... 2<br>Housewife/Househusband .... 3<br>Unemployed .... 4<br>Student ……………………………………...5	

SD9	IN YOUR CURRENT RESIDENCE, HOW MANY ROOMS DO YOU HAVE FOR THE USE OF YOU AND YOUR FAMILY?	Rooms .... __ __	
SD10	HOW MANY ROOMS ARE USED FOR SLEEPING?	Rooms .... __ __	
SD11	HOW MANY PEOPLE, INCLUDING YOURSELF, USUALLY SLEEP IN YOUR HOUSEHOLD?	People .... __ __	
SD12	WHAT TYPE OF FUEL DOES YOUR HOUSEHOLD MAINLY USE FOR COOKING?	Electricity .... 1<br>Bottled gas/Biogas .... 2<br>Paraffin/Kerosene .... 3<br>Charcoal .... 4<br>Firewood.. 5<br>Solar .... 6<br>Other .... 7<br>*(specify)* ___________________________	
SD13	WHAT IS THE MAIN SOURCE OF ENERGY FOR LIGHTING IN YOUR HOUSEHOLD?	Solar .... 1<br>Gas .... 2<br>Paraffin/Hurricane lamp............................. 3<br>Paraffin/Pressure lamp .... 4<br>Paraffin wick lamp .... 5<br>Electricity ……………………………………6<br>Other .... 7<br>*(specify)* ___________________________	
SD14	WHAT IS THE MAIN MATERIAL OF THE FLOOR IN YOUR HOUSEHOLD?	Earth and/or sand...................................... 1<br>Dung .... 2<br>Rudimentary floor .... 3<br>Wooden floor .... 4<br>Vinyl or asphalt strips .... 5<br>Cement screed .... 6<br>Tiles.. 7<br>Other .... 8<br>*(specify)* ___________________________	
SD15	WHAT IS THE MAIN MATERIAL OF THE WALL IN YOUR HOUSEHOLD?	Poles and mud... 1<br>Sundried bricks .... 2<br>Baked bricks .... 34<br>Timber .... 4<br>Cement bricks... 5<br>Stones... 6<br>Other .... 7<br>*(specify)* ___________________________	

SD16	WHAT IS THE MAIN MATERIAL OF THE ROOF IN YOUR HOUSEHOLD?	Grass/Leaves/ .... 1<br>Iron sheets .... 2<br>Tiles .... 3<br>Concrete .... 4<br>Other .... 5<br>*(specify)* ________________________	
SD17	WHAT IS THE MAIN SOURCE OF DRINKING WATER FOR YOUR HOUSEHOLD?	Piped into dwelling/yard/plot .... 1<br>Public/Neighbour's tap............................... 2<br>Open well in dwelling/yard/plot .... 3<br>Open public/neighbour's well .... 4<br>Protected well in dwelling/yard/plot .... 5<br>Protected public/neighbour well .... 6<br>Spring .... 7<br>River, stream .... 8<br>Pond/Lake/Dam... 9<br>Tanker truck .... 10<br>Water vendor .... 11<br>Other .... 12<br>*(specify)* ________________________	
SD18	HOW OFTEN IN THE LAST 6 MONTHS DID YOUR HOUSEHOLD HAVE PROBLEMS IN SATISFYING THEIR FOOD NEEDS?	Never………………………………………1<br>Seldom ……………………………………....2<br>Sometimes…………………………………3<br>Often…………………………………………4<br>Always………………………………………5	
SD19	WHEN YOU ARE IN YOUR HOUSEHOLD, WHAT KIND OF TOILET FACILITY DO YOU USUALLY USE?	Flush toilet used by household/family only 1<br>Pit latrine used by household/family only .. 2<br>Flush toilet shared with other tenants........ 3<br>Pit Latrine shared with other tenants......... 4<br>Flush toilet shared with a neighbor in<br>different house .... 5<br>No facility/Bush/Field 7	
SD20	HOW MANY CHILDREN (AGED UNDER 18 YEARS) ARE CURRENTLY LIVING IN YOUR HOUSEHOLD?	Children .... __ __	

"I would like to ask you some questions about your knowledge of HIV/AIDS."

SECTION 2: HIV/AIDS KNOWLEDGE

HK1	CAN PEOPLE REDUCE THEIR CHANCE OF GETTING THE AIDS VIRUS BY HAVING JUST ONE UNINFECTED SEX PARTNER WHO HAS NO OTHER SEX PARTNERS?	Yes....1<br>No ....2<br><br>Don't know 88	
HK 2	CAN PEOPLE GET THE AIDS VIRUS FROM MOSQUITO BITES?	Yes....1<br>No ....2<br><br>Don't know 88	
HK 3	CAN PEOPLE REDUCE THEIR CHANCE OF GETTING THE AIDS VIRUS BY USING A CONDOM EVERY TIME THEY HAVE SEX?	Yes....1<br>No ....2<br><br>Don't know 88	
HK 4	CAN PEOPLE GET THE AIDS VIRUS BY SHARING FOOD WITH A PERSON WHO HAS AIDS?	Yes....1<br>No ....2<br><br>Don't know 88	
HK 5	CAN PEOPLE REDUCE THEIR CHANCE OF GETTING THE AIDS VIRUS BY NOT HAVING SEXUAL INTERCOURSE AT ALL?	Yes....1<br>No ....2<br><br>Don't know 88	
HK 6	CAN PEOPLE GET THE AIDS VIRUS BECAUSE OF WITCHCRAFT OR OTHER SUPERNATURAL MEANS?	Yes....1<br>No ....2<br><br>Don't know 88	
HK 7	IS IT POSSIBLE FOR A HEALTHY-LOOKING PERSON TO HAVE THE AIDS VIRUS?	Yes....1<br>No ....2<br><br>Don't know 88	
HK 8	IF A MAN HAS THE VIRUS THAT CAUSES AIDS DOES HIS SEXUAL PARTNER ALWAYS, ALMOST ALWAYS, OR ONLY SOMETIMES HAVE THE AIDS VIRUS?	Always ....1<br>Almost always....2<br>Sometimes....3<br><br>Don't know 88	

HK 9	IF A WOMAN HAS THE VIRUS THAT CAUSES AIDS DOES HER SEXUAL PARTNER ALWAYS, ALMOST ALWAYS, OR ONLY SOMETIMES HAVE THE AIDS VIRUS?	Always ....1<br>Almost always ....2<br>Sometimes ....3<br><br>Don't know 88	
HK 10	CAN THE VIRUS THAT CAUSES AIDS BE TRANSMITTED FROM A MOTHER TO HER BABY?<br><br>IF NO, SKIP TO HK11.	Yes ....1<br>No ....2<br><br>Don't know 88	
HK 10.1	DURING PREGNANCY?	Yes ....1<br>No ....2<br><br>Don't know 88	
HK 10.2	DURING DELIVERY?	Yes ....1<br>No ....2<br><br>Don't know 88	
HK 10.3	BY BREASTFEEDING?	Yes ....1<br>No ....2<br><br>Don't know 88	
HK 11.1	ARE THERE SPECIAL DRUGS THAT A DOCTOR OR A NURSE CAN GIVE TO A WOMAN INFECTED WITH THE AIDS VIRUS TO REDUCE THE RISK OF TRANSMISSION TO THE BABY?	Yes ....1<br>No ....2<br><br>Don't know 88	

HK11.2	HAVE YOU HEARD ABOUT SPECIAL ANTIRETROVIRAL DRUGS (USE LOCAL NAME) THAT PEOPLE INFECTED WITH THE VIRUS CAN GET FROM A DOCTOR OR A NURSE TO HELP THEM LIVE LONGER?	Yes .... 1<br>No 2	

HK 12	WHAT ARE THE MAIN CHANNELS OF COMMUNICATION FROM WHICH YOU RECEIVE AIDS INFORMATION AND EDUCATION?<br><br>PROBE: ANY OTHER CHANNELS?<br><br>[SELECT ALL THAT APPLY.]	Radio<br>Yes ....1<br>No ....2<br><br>Television<br>Yes ....1<br>No ....2<br><br>Film<br>Yes ....1<br>No ....2<br><br>Drama<br>Yes ....1<br>No ....2<br><br>Newspapers/magazines<br>Yes ....1<br>No ....2<br><br>Brochures<br>Yes ....1<br>No ....2<br><br>Posters<br>Yes ....1<br>No ....2<br><br>Billboards<br>Yes ....1<br>No ....2<br><br>Community notices<br>Yes ....1<br>No ....2<br><br>Family<br>Yes ....1<br>No ....2<br><br>Friends<br>No ....2<br>Yes ....1<br><br>Peers<br>No ....2<br>Yes ....1<br><br>Health workers<br>No ....2<br>Yes ....1<br><br>Teachers<br>[illegible]	

(HK 12)	WHAT ARE THE <u>MAIN</u> CHANNELS OF COMMUNICATION FROM WHICH YOU RECEIVE AIDS INFORMATION AND EDUCATION?<br><br>(PROBE: ANY OTHER CHANNELS?)	Traditional leaders<br>Yes .... 1<br>No .... 2<br><br>Religious leaders<br>Yes .... 1<br>No .... 2<br><br>Internet<br><br>Yes .... 1<br>No .... 2<br><br>Other<br>Yes .... 1<br>No .... 2<br><br>(specify) _____________<br><br>None<br>Yes .... 1<br>No .... 2<br><br>Refused........77	

"Now I am going to read some statements, please tell me whether you strongly agree, agree, are neutral, disagree, or strongly disagree."

SECTION 3: BELIEFS AND PERCEPTIONS REGARDING EFFECT OF ANTI RETROVIRALS ON TRANSMISSION AND PREVENTION OF HIV/AIDS			
ARV 1	I AM LESS CONCERNED ABOUT INFECTING SOMEONE BECAUSE OF THE AVAILABILITY OF ANTIRETROVIRAL DRUGS (ARVS).	Strongly disagree .... 1<br>Disagree .... 2<br>Neutral .... 3<br>Agree .... 4<br>Strongly Agree 5	
ARV 2	BECAUSE ANTIRETROVIRAL DRUGS (ARVS) ARE BECOMING INCREASINGLY AVAILABLE, I THINK THERE IS NO LONGER A NEED FOR REDUCING THE NUMBER OF PARTNERS.	Strongly disagree .... 1<br>Disagree .... 2<br>Neutral .... 3<br>Agree .... 4<br>Strongly Agree 5	

ARV 3	BECAUSE ANTIRETROVIRAL DRUGS (ARVS) ARE BECOMING INCREASINGLY AVAILABLE, I THINK THERE IS NO LONGER A NEED FOR USING CONDOMS.	Strongly disagree .... 1<br>Disagree .... 2<br>Neutral .... 3<br>Agree .... 4<br>Strongly Agree 5	
ARV 4	MAKING DECISIONS ABOUT WHO BEST TO TELL MAKES IT POSSIBLE TO CHOOSE WHEN TO TELL THAT PERSON ABOUT MY HIV STATUS	Strongly disagree .... 1<br>Disagree .... 2<br>Neutral .... 3<br>Agree .... 4<br>Strongly Agree 5	
ARV 5	I THINK TELLING A SEXUAL PARTNER THAT I HAVE HIV, INCREASES THEIR SUPPORT FOR MY USE OF ART DRUGS	Strongly disagree .... 1<br>Disagree .... 2<br>Neutral .... 3<br>Agree .... 4<br>Strongly Agree 5	
ARV 7	WHEN USING ART DRUGS, I GET MORE SUPPORT IF I SHARE MY HIV STATUS WITH A FAMILY MEMBER OR A FRIEND	Strongly disagree .... 1<br>Disagree .... 2<br>Neutral .... 3<br>Agree .... 4<br>Strongly Agree 5	
ARV 8	WHEN I AM USING ART DRUGS, I DO NOT NEED TO TELL MY SEXUAL PARTNER ABOUT MY HIV STATUS	Strongly disagree .... 1<br>Disagree .... 2<br>Neutral .... 3<br>Agree .... 4<br>Strongly Agree 5	
ARV 9	LISTENING to AND UNDERSTANDING MY PARTNER DECREASES CONFLICT WHEN TALKING ABOUT HIV	Strongly disagree .... 1<br>Disagree .... 2<br>Neutral .... 3<br>Agree .... 4<br>Strongly Agree 5	

"I will ask a few questions to get your thoughts about living with HIV. Please indicate the extent to which you agree and disagree with each statement."

**SECTION 4: HIV STIGMA AND DISCRIMINATION**			
HSD 1	I FEEL GUILTY THAT I AM HIV POSITIVE.	Disagree strongly .... 1<br>Disagree .... 2<br>Neutral .... 3<br>Agree .... 4<br>Agree strongly 5	

HSD 2	I FEEL ASHAMED THAT I AM HIV POSITIVE.	Disagree strongly ..... 1<br>Disagree slightly ..... 2<br>Neutral ..... 3<br>Agree slightly ..... 4<br>Agree strongly 5	
HSD 3	PEOPLE STILL HUG ME EVEN THOUGH THEY KNOW I HAVE HIV.<br><br>[N/A IF YOU HAVE NOT TOLD ANYONE YOU HAVE HIV]	Disagree strongly ..... 1<br>Disagree slightly ..... 2<br>Neutral ..... 3<br>Agree slightly ..... 4<br>Agree strongly ..... 5<br><br>N/A.....99	
HSD 4	PEOPLE I CARE ABOUT CALL ME MORE OFTEN AFTER THEY LEARNED THAT I HAVE HIV.<br><br>[N/A IF YOU HAVE NOT TOLD ANYONE YOU HAVE HIV]	Disagree strongly ..... 1<br>Disagree slightly ..... 2<br>Neutral ..... 3<br>Agree slightly ..... 4<br>Agree strongly ..... 5<br><br>N/A.....99	
HSD 5	PEOPLE ARE HAPPY TO SPEND TIME WITH ME KNOWING I HAVE HIV.<br><br>[N/A IF YOU HAVE NOT TOLD ANYONE YOU HAVE HIV]	Disagree strongly ..... 1<br>Disagree slightly ..... 2<br>Neutral ..... 3<br>Agree slightly ..... 4<br>Agree strongly ..... 5<br><br>N/A.....99	
HSD 6	PEOPLE HAVE AVOIDED ME BECAUSE I HAVE HIV.<br><br>[N/A IF YOU HAVE NOT TOLD ANYONE YOU HAVE HIV]	Disagree strongly ..... 1<br>Disagree slightly ..... 2<br>Neutral ..... 3<br>Agree slightly ..... 4<br>Agree strongly ..... 5<br><br>N/A.....99	
HSD 7	PEOPLE LET ME PLAY WITH THEIR CHILDREN ALTHOUGH THEY KNOW I HAVE HIV.<br><br>[N/A IF YOU HAVE NOT TOLD ANYONE YOU HAVE HIV]	Disagree strongly ..... 1<br>Disagree slightly ..... 2<br>Neutral ..... 3<br>Agree slightly ..... 4<br>Agree strongly ..... 5<br><br>N/A.....99	

"Here is a list of some things that other people do for us or give us that may be helpful or supportive. Please indicate the response that is closest to your situation."

**SECTION 5: SOCIAL SUPPORT**			
SS1	I GET VISITS FROM FRIENDS AND RELATIVES.	As much as I would like ..... 1<br>Less than I would like ..... 2<br>Much less than I would like ..... 3<br>Never 4	

SS 2	I GET USEFUL ADVICE ABOUT IMPORTANT THINGS IN LIFE.	As much as I would like .... 1<br>Less than I would like .... 2<br>Much less than I would like .... 3<br>Never 4	
SS 3	I GET CHANCES TO TALK TO SOMEONE ABOUT PROBLEMS AT WORK OR WITH MY HOUSEWORK.	As much as I would like .... 1<br>Less than I would like .... 2<br>Much less than I would like .... 3<br>Never 4	
SS 4	I GET CHANCES TO TALK TO SOMEONE I TRUST ABOUT MY PERSONAL AND FAMILY PROBLEMS.	As much as I would like .... 1<br>Less than I would like .... 2<br>Much less than I would like .... 3<br>Never 4	
SS 5	I GET HELP AROUND THE HOUSE.	As much as I would like .... 1<br>Less than I would like .... 2<br>Much less than I would like .... 3<br>Never 4	
SS 6	I GET HELP WITH MONEY IN AN EMERGENCY.	As much as I would like .... 1<br>Less than I would like .... 2<br>Much less than I would like .... 3<br>Never 4	
SS 7	I GET HELP WHEN I NEED TRANSPORTATION.	As much as I would like .... 1<br>Less than I would like .... 2<br>Much less than I would like .... 3<br>Never 4	
SS 8	I GET HELP WHEN I AM SICK.	As much as I would like .... 1<br>Less than I would like .... 2<br>Much less than I would like .... 3<br>Never 4	
SS 9	I HAVE PEOPLE WHO CARE WHAT HAPPENS TO ME.	As much as I would like .... 1<br>Less than I would like .... 2<br>Much less than I would like .... 3<br>Never 4	
SS 10	I GET LOVE AND AFFECTION.	As much as I would like .... 1<br>Less than I would like .... 2<br>Much less than I would like .... 3<br>Never 4	

“Please indicate how confident you are that you could perform each of the following tasks.”

SECTION 6: SELF- EFFICACY FOR SAFE SEX, DISCLOSURE, AND PREVENTION ADVOCACY

SES 1	**HOW CONFIDENT ARE YOU THAT YOU** KNOW WHERE TO GET CONDOMS WHEREVER YOU ARE?	Not at all confident……………………….1<br>Not very confident .... 2<br>Somewhat confident .... 3<br>Very confident……………………………... 4	
SES 2	**HOW CONFIDENT ARE YOU THAT YOU** MAKE SURE THAT YOU HAVE CONDOMS WITH YOU AT ALL TIMES?	Not at all confident……………………….1<br>Not very confident .... 2<br>Somewhat confident .... 3<br>Very confident……………………………... 4	
SES 3	**HOW CONFIDENT ARE YOU THAT YOU** CAN USE CONDOMS CORRECTLY AND CONSISTENTLY WHEN YOU HAVE HAD A DRINK?	Not at all confident……………………….1<br>Not very confident .... 2<br>Somewhat confident .... 3<br>Very confident……………………………... 4	
SES 4	**HOW CONFIDENT ARE YOU THAT YOU CAN** REFUSE TO HAVE SEX WITH YOUR PARTNER IF HE/SHE DOES NOT WANT TO USE CONDOMS?	Not at all confident……………………….1<br>Not very confident .... 2<br>Somewhat confident .... 3<br>Very confident……………………………... 4	
SES 5	**HOW CONFIDENT ARE YOU THAT YOU** CAN TALK WITH YOUR PARTNER(S) ABOUT THE IMPORTANCE OF USING CONDOMS WHEN YOU HAVE SEX?	Not at all confident……………………….1<br>Not very confident .... 2<br>Somewhat confident .... 3<br>Very confident……………………………... 4	
SES 6	**HOW CONFIDENT ARE YOU THAT YOU** CAN TALK WITH FAMILY AND/OR FRIENDS ABOUT HOW TO PROTECT AGAINST HIV INFECTION?	Not at all confident……………………….1<br>Not very confident .... 2<br>Somewhat confident .... 3<br>Very confident……………………………... 4	

SES 7	**HOW CONFIDENT ARE YOU THAT YOU CAN** TALK WITH FAMILY AND/OR FRIENDS ABOUT THE IMPORTANCE OF USING CONDOMS CORRECTLY EVERY TIME THEY ARE HAVING SEX?	Not at all confident……………………….1<br>Not very confident .... 2<br>Somewhat confident .... 3<br>Very confident…………………………….... 4	
SES 8	**HOW CONFIDENT ARE YOU THAT YOU** CAN TALK WITH FAMILY AND/OR FRIENDS ABOUT THE IMPORTANCE OF GETTING TESTED FOR HIV?	Not at all confident……………………….1<br>Not very confident .... 2<br>Somewhat confident .... 3<br>Very confident…………………………….... 4	
SES 9	**HOW CONFIDENT ARE YOU THAT YOU** CAN TALK WITH FAMILY AND/OR FRIENDS ABOUT SEEKING HIV CARE IF THEY HAVE HIV?	Not at all confident……………………….1<br>Not very confident .... 2<br>Somewhat confident .... 3<br>Very confident…………………………….... 4	
SES 10	**HOW CONFIDENT ARE YOU THAT YOU** CAN TALK WITH FAMILY AND/OR FRIENDS ABOUT THE IMPORTANCE OF DISCLOSING TO OTHERS ABOUT THEIR HIV STATUS?	Not at all confident……………………….1<br>Not very confident .... 2<br>Somewhat confident .... 3<br>Very confident…………………………….... 4	
SES 11	**HOW CONFIDENT ARE YOU THAT YOU** CAN MAKE DECISIONS ABOUT WHEN IT IS THE RIGHT TIME TO TELL SOMEONE THAT YOU HAVE HIV?	Not at all confident……………………….1<br>Not very confident .... 2<br>Somewhat confident .... 3<br>Very confident…………………………….... 4	
SES 12	**HOW CONFIDENT ARE YOU THAT YOU** CAN MAKE GOOD DECISIONS ABOUT WHO TO TELL THAT YOU HAVE HIV?	Not at all confident……………………….1<br>Not very confident .... 2<br>Somewhat confident .... 3<br>Very confident…………………………….... 4	

SES 13	HOW CONFIDENT ARE YOU THAT YOU CAN FOLLOW THROUGH WITH TELLING SOMEONE YOU HAVE HIV, ONCE YOU HAVE MADE THE DECISION THAT YOU WANT TO TELL THAT PERSON?	Not at all confident……………………….1<br>Not very confident .... 2<br>Somewhat confident .... 3<br>Very confident……………………………... 4	

"Please indicate how confident you are that you could perform each of the following tasks."

**SECTION 7: THE GENERAL SELF-EFFICACY SCALE**			
GSE 1	IF SOMEONE OPPOSES ME, I CAN FIND THE MEANS AND WAYS TO GET WHAT I WANT.	Not at all true .... 1<br>Hardly true .... 2<br>Moderately true .... 3<br>Exactly true 4	
GSE 2	I AM CONFIDENT THAT I COULD DEAL EFFICIENTLY WITH UNEXPECTED EVENTS.	Not at all true .... 1<br>Hardly true .... 2<br>Moderately true .... 3<br>Exactly true 4	
GSE 3	THANKS TO MY RESOURCEFULNESS, I KNOW HOW TO HANDLE UNFORESEEN SITUATIONS.	Not at all true .... 1<br>Hardly true .... 2<br>Moderately true .... 3<br>Exactly true 4	
GSE 4	WHEN I AM CONFRONTED WITH A PROBLEM, I CAN USUALLY FIND SEVERAL SOLUTIONS.	Not at all true .... 1<br>Hardly true .... 2<br>Moderately true .... 3<br>Exactly true 4	
GSE 5	IF I AM IN TROUBLE, I CAN USUALLY THINK OF A SOLUTION.	Not at all true .... 1<br>Hardly true .... 2<br>Moderately true .... 3<br>Exactly true 4	

"Now I am going to ask you some personal questions about your life. This information will be kept secret; nobody here at the clinic, including the nurses and doctors, will see your responses. If we should come to a question that you do not want to answer, just let me know and we will go on to the next question."

SECTION 8: SEXUAL RELATIONSHIPS

SR1	AT WHAT AGE DID YOU FIRST HAVE SEXUAL INTERCOURSE?	YEARS _ _	
SR2	HAVE YOU HAD SEXUAL INTERCOURSE IN THE LAST 6 MONTHS?	YES 1<br>NO 2<br>IF NO⇨ SKIP TO NEXT SECTION	
SR3	HOW MANY SEXUAL PARTNERS HAVE YOU HAD IN THE LAST 6 MONTHS?	NUMBER OF SEXUAL PARTNERS __ ___	

SR	4. WHEN DID YOU FIRST START A RELATIONSHIP WITH THIS PARTNER?	5. WHAT IS THIS PARTNER'S RELATIONSHIP TO YOU [READ ALL RESPONSE CHOICES]?	6. HOW OFTEN HAVE YOU HAD SEX WITH THIS PERSON IN THE PAST 6 MONTHS?	7. HOW OFTEN DID YOU USE CONDOMS WITH THIS PERSON IN THE PAST 6 MONTHS?	8. HOW MANY TIMES DID YOU HAVE SEX WITH THIS PARTNER IN THE PAST 30 DAYS?	9. HOW MANY TIMES DID YOU USE A CONDOM DURING SEX WITH THIS PARTNER IN THE PAST 30 DAYS?		10. WHY DID YOU NOT USE A CONDOM WITH THIS PARTNER AT TIMES?	11. WHEN DID YOU FIRST HAVE SEX WITH THIS PARTNER?	12. WHEN DID YOU LAST HAVE SEX WITH THIS PARTNER?	13. DO YOU PLAN TO HAVE SEX WITH THIS PERSON AGAIN?	14.WHAT IS THE HIV STATUS OF THIS PARTNER?	15. HAVE YOU SHARED YOUR HIV STATUS WITH THIS PARTNER?	16. HAVE YOU HAD SEX WITH ANY OTHER PARTNER IN THE LAST 6 MONTHS?
P1	Year _ _<br>Month _ _<br>Day ____<br>Refused…..77<br>N/A……..99	Spouse ………..1<br>Cohabitating partner…2<br>Non-cohabitating regular partner….3<br>Casual partner (friend/other)....4<br>Commercial sex worker/ client…...... …...5<br>Other (specify)….6<br>*(specify)*<br>______________<br>Refused. …….77	Only once ….1<br>More than once but less than monthly.........2<br>About once per month………3<br>More than once per month but less than weekly……...4<br>Weekly……..5	Never…..1<br>Some of the time …….2<br>All of the time…….3<br>Refused..77	Times _ _<br>If 00 ⇨ SKIP Q9<br>Refused..77	Times _ _<br>Refused...77<br>N/A ……99	CHECK for unprotected sex.<br>If Q7=3 AND Q8=Q9 SKIP to Q11<br>Else go to Q10	Both HIV positive .... …1<br>Not Available......2<br>Too Expensive.. 3<br>Partner objected …...4<br>Don't like them. ....5<br>Used other contraceptive6<br>Didn't think it was necessary..7<br>Didn't think of it 8	Year…………<br>Month……….<br>Day………….<br>Refused….77	Year _ _<br>Month _ _<br>Day ___<br>Refused..77	No……2<br>Yes…..1<br>Don't know ...88<br>Refused.77	Positive. ....1<br>Negative ....2<br>Don't know ..88	No……2<br>Yes…..1<br>Refused..77	No........2<br>If No ⇨ SKIP to Next Section<br>Yes ......1<br>If Yes ⇨ Repeat questions for next partner<br>Refused ....77<br>If Refused ⇨ SKIP to Next

			Refused……77					Other (please specify) ....9<br>Refused 77						Section
P2	Year _ _<br>Month _ _<br>Day ____<br><br>Refused…..77<br>N/A……..99	Spouse ………..1<br>Cohabitating partner…2<br>Non-cohabitating regular partner….3<br>Casual partner (friend/other) ....4<br>Commercial sex worker/ client…..... …...5<br>Other (specify)….6<br>*(specify)*<br>____________<br><br>Refused. …….77	Only once ….1<br><br>More than once but less than monthly.........2<br><br>About once per month………3<br><br>More than once per month but less than weekly……...4<br><br>Weekly……..5<br><br>Refused……77	Never…..1<br><br>Some of the time …….2<br><br>All of the time…….3<br><br>Refused..7	Times _ _<br><br>If 00 ⇨ SKIP Q9<br><br>Refused..77	Times _ _<br><br>Refused...7<br>N/A ……...9	CHECK for unprotected sex.<br><br>If Q7=3 AND Q8=Q9 SKIP to Q11<br><br>Else go to Q10	Both HIV positive .... …1<br>Not Available......2<br>Too Expensive.. 3<br>Partner objected …...4<br>Don't like them. ....5<br>Used other contraceptive6<br>Didn't think it was necessary..10<br>Didn't think of it ....11<br>Other (please specify) ......12<br>Refused 77	Year………..<br>Month………<br>Day…………<br>Refused….77	Year _ _<br>Month _ _<br>Day ___<br><br>Refused..77	No……2<br>Yes…..1<br>Don't know ...8<br><br>Refused.77	Positive. ....1<br>Negative ....2<br>Don't know ..88	No……2<br>Yes…..1<br><br>Refused..77	No ....2<br>If No ⇨ SKIP to Next Section<br><br>Yes ......1<br>If Yes ⇨ Repeat questions for next partner<br><br>Refused ....77<br>If Refused ⇨ SKIP to Next Section
P3	Year _ _<br>Month _ _<br>Day ____<br><br>Refused…..77<br>N/A……..99	Spouse ………..1<br>Cohabitating partner…2<br>Non-cohabitating regular partner….3<br>Casual partner (friend/other) ....4<br>Commercial sex worker/ client…..... …...5<br>Other (specify)….6<br>*(specify)*<br>____________<br><br>Refused. …….77	Only once ….1<br><br>More than once but less than monthly.........2<br><br>About once per month………3<br><br>More than once per month but less than weekly……...4	Never…..1<br><br>Some of the time …….2<br><br>All of the time…….3<br><br>Refused..7	Times _ _<br><br>If 00 ⇨ SKIP Q9<br><br>Refused..77	Times _ _<br><br>Refused...7<br>N/A ……...9	CHECK for unprotected sex.<br><br>If Q7=3 AND Q8=Q9 SKIP to Q11<br><br>Else go to Q10	Both HIV positive .... …1<br>Not Available......2<br>Too Expensive.. 3<br>Partner objected …...4<br>Don't like them. ....5<br>Used other contraceptive6<br>Didn't think it was necessary..10	Year………..<br>Month………<br>Day…………<br>Refused….77	Year _ _<br>Month _ _<br>Day ___<br><br>Refused..77	No……2<br>Yes…..1<br>Don't know ...8<br><br>Refused.77	Positive. ....1<br>Negative ....2<br>Don't know ..88	No……2<br>Yes…..1<br><br>Refused..77	No ....2<br>If No ⇨ SKIP to Next Section<br><br>Yes ......1<br>If Yes ⇨ Repeat questions for next partner<br><br>Refused ....77<br>If

			Weekly……..5<br>Refused……77					Didn't think of it ....11<br>Other (please specify) ......12<br>Refused 77						Refused ⇨ SKIP to Next Section
P4	Year _ _<br>Month _ _<br>Day ____<br>Refused…..77<br>N/A……..99	Spouse ………..1<br>Cohabitating partner…2<br>Non-cohabitating regular partner….3<br>Casual partner (friend/other) ....4<br>Commercial sex worker/ client…..... …...5<br>Other (specify)….6<br>*(specify)*<br>____________<br>Refused. …….77	Only once ….1<br>More than once but less than monthly.........2<br>About once per month………3<br>More than once per month but less than weekly……...4<br>Weekly……..5<br>Refused……77	Never…..1<br>Some of the time …….2<br>All of the time…….3<br>Refused..7	Times _ _<br>If 00 ⇨ SKIP Q9<br>Refused..77	Times _ _<br>Refused...7<br>N/A ……...9	CHECK for unprotected sex.<br>If Q7=3 AND Q8=Q9 SKIP to Q11<br>Else go to Q10	Both HIV positive .... …1<br>Not Available ......2<br>Too Expensive .. 3<br>Partner objected ......4<br>Don't like them. ....5<br>Used other contraceptive6<br>Didn't think it was necessary..10<br>Didn't think of it ....11<br>Other (please specify) ......12<br>Refused 77	Year………..<br>Month………<br>Day…………<br>Refused….77	Year _ _<br>Month _ _<br>Day ___<br>Refused..77	No……2<br>Yes…..1<br>Don't know ...8<br>Refused.77	Positive. ....1<br>Negative ....2<br>Don't know ..88	No……2<br>Yes…..1<br>Refused..77	No ....2<br>If No ⇨ SKIP to Next Section<br>Yes ......1<br>If Yes ⇨ Repeat questions for next partner<br>Refused ....77<br>If Refused ⇨ SKIP to Next Section

SR17. OTHER THAN THE PARTNERS YOU'VE DESCRIBED IN DETAIL ABOVE, HOW MANY ADDITIONAL PARTNERS IN THE PAST 6 MONTHS DID YOU HAVE?	NUMBER OF SEXUAL PARTNERS __ ___<br>IF 0 SKIP TO 8A	

SR.18. IF YOU HAVE ANY OTHER PARTNERS, HAVE YOU DISCLOSED YOUR HIV STATUS TO THEM?	YES, FOR ALL OF THEM………….1<br>YES, FOR SOME OF THEM………..2<br>NO……………………………….3<br>REFUSED……………………… 77<br><br>N/A, I DON'T HAVE ANY OTHER SEXUAL PARTNERS………………………..99	

8a. Have you been involved in an intimate relationship, such as marriage, living with someone, or dating within the past 6 months?

If YES, go to Q. IPV1

If NO, skip to Section 10.

"Now, if you will permit me, I need to ask you some questions about your relationship with your primary/main intimate partner/husband/wife. If we should come to a question that you do not want to answer, just let me know and we will go on to the next question."

SECTION 09: INTIMATE PARTNER VIOLENCE			
IPV 1	HOW MUCH DO YOU APPRECIATE THE RELATIONSHIP WITH YOUR PARTNER?	Not at all .... 1<br>A little .... 2<br>Somewhat .... 3<br>Quite a lot .... 4<br>Very much .... 5<br><br>Refused to answer .... 77<br>N/A 99	
IPV 2	DO YOU FEEL THAT YOU ARE APPRECIATED BY YOUR PARTNER?	Not at all .... 1<br>A little .... 2<br>Somewhat .... 3<br>Quite a lot .... 4<br>Very much .... 5<br><br>Refused to answer .... 77<br>N/A 99	
IPV 3	DO YOU FEEL THAT YOUR PARTNER LISTENS TO YOU?	Not at all .... 1<br>A little .... 2<br>Somewhat .... 3<br>Quite a lot .... 4<br>Very much .... 5<br><br>Refused to answer .... 77<br>N/A 99	

NOW, I WILL ASK ABOUT DIFFERENT SITUATIONS THAT EXIST IN INTIMATE RELATIONSHIPS. PLEASE TELL ME IF ANY OF THE FOLLOWING HAS HAPPENED IN THE RELATIONSHIP WITH YOUR PARTNER IN THE LAST 6 MONTHS.

A1) WAS JEALOUS OR ANGRY IF YOU TALKED WITH …<br>(FEMALE RESPONDENT) ….PERSONS OF THE OPPOSITE SEX?	*IPV4A*	NO 2<br>YES 1	A2) WERE YOU EVER JEALOUS OR ANGRY IF YOUR PARTNER TALKED WITH PERSONS OF THE OPPOSITE SEX?	*IPV4B*	NO 2<br>YES 1
B1) OFTEN SUSPICIOUS THAT YOU ARE UNFAITHFUL?	*IPV5A*	NO 2<br>YES 1	B2) WERE YOU OFTEN SUSPICIOUS THAT YOUR PARTNER WAS UNFAITHFUL?	*IPV5B*	NO 2<br>YES 1
C1) DID NOT ALLOW YOU TO MEET WITH FRIENDS?	*IPV6A*	NO 2<br>YES 1	C2) DID YOU ALLOW YOUR PARTNER TO MEET WITH FRIENDS?	*IPV6B*	NO 2<br>YES 1
D1) PUT LIMITS TO YOUR COMMUNICATION WITH YOUR FAMILY?	*IPV7A*	NO 2	D2) DID YOU PUT LIMITS TO YOUR PARTNERS COMMUNICATION WITH HIS/HER FAMILY?	*IPV7B*	NO 2

		YES 1			YES 1
E1) INSISTED TO KNOW WHERE YOU WERE EVERY MOMENT?	*IPv8A*	NO 2<br>YES 1	E2) DID YOU INSIST TO KNOW WHERE YOUR PARTNER IS EVERY MOMENT?	*IPV8B*	NO 2<br>YES 1
F1) DID NOT TRUST YOU WITH ANYTHING RELATED TO MONEY?	*IPV9A*	NO 2<br>YES 1	F2) DID YOU TRUST YOUR PARTNER WITH ANYTHING RELATED TO MONEY?	*IPV9B*	NO 2<br>YES 1

IF YOU WILL ALLOW ME, I WOULD LIKE TO ASK MORE QUESTIONS ABOUT YOUR RELATIONSHIP WITH YOUR INTIMATE PARTNER . IF I ASK A QUESTION YOU DO NOT WISH TO RESPOND TO, PLEASE INFORM ME SO THAT I CAN ASK THE FOLLOWING QUESTION.

G1. HAS YOUR INTIMATE PARTNER: IN THE PAST 6 MONTHS EVER:			G2 HAVE YOU IN THE PAST 6 MONTHS EVER:		
G1A) SAID OR DONE ANYTHING IN THE PRESENCE OF OTHER PEOPLE THAT EMBARRASSED OR SHAMED YOU?	*IPV10A*	NO 2<br>YES 1	G2A) SAID OR DONE ANYTHING IN THE PRESENCE OF OTHER PEOPLE THAT EMBARRASSED OR SHAMED YOUR HUSBAND/WIFE OR INTIMATE PARTNER?	*IPV10B*	NO 2<br>YES 1
G1B) THREATENED TO HURT YOU/ OR SOMEONE THAT IS CLOSE TO YOU?	*IPV11A*	NO 2<br>YES 1	G2B) THREATENED TO HURT Y YOUR HUSBAND/WIFE OR INTIMATE PARTNER/OR SOMEONE CLOSE TO THEM?	*IPV11B*	NO 2<br>YES 1
G1C) INSULTED YOU OR MADE YOU FEEL BAD ABOUT YOURSELF?	*IPV12A*	NO 2<br>YES 1	G2C) INSULTED YOU OR MADE YOUR HUSBAND/WIFE OR INTIMATE PARTNER FEEL BAD ABOUT THEMSELVES?	*IPV12B*	NO 2<br>YES 1

H1. HAS YOUR HUSBAND/WIFE OR INTIMATE PARTNER (EVER DONE ANY OF THE FOLLOWING IN THE PAST 6 MONTHS?			H2. HAVE YOU EVER DONE ANY OF THE FOLLOWING IN THE PAST 6 MONTHS TO YOUR INTIMATE PARTNER		
H1A) PUSHED, SHOOK OR OR THREW SOMETHING AT YOU?	*IPV13 A*	NO 2<br>YES 1	H2A) PUSHED, SHOOK OR OR THREW SOMETHING AT YOUR PARTNER?	*IPV13 B*	NO 2<br>YES 1
H1B) SLAPPED YOU?	*IPV14 A*	NO 2<br>YES 1	H2B) SLAPPED YOUR PARTNER?	*IPV14 B*	NO 2<br>YES 1
H1C) TWISTED YOUR ARM OR PULLED YOUR HAIR?	*IPV15A*	NO 2<br>YES 1	H2C) TWISTED ARM OR PULLED HAIR OFYOUR PARTNER?	*IPV15 B*	NO 2<br>YES 1
H1D) HIT WITH FIST OR SOMETHING ELSE THAT COULD HURT YOU?	*IPV16A*	NO 2<br>YES 1	H2D) HIT WITH FIST OR SOMETHING ELSE THAT COULD HURT YOUR PARTNER?	*IPV16 B*	NO 2<br>YES 1
H1E) KICKED, DRAGGED ACROSS SURFACE OR BEAT YOU?	*IPV17A*	NO 2<br>YES 1	H2E) KICKED, DRAGGED ACROSS SURFACE OR BEAT PARTNER?	*IPV17 B*	NO 2<br>YES 1
H1F) TRIED TO CHOKE YOU OR BURN/SCALD YOU ON PURPOSE?	*IPV18A*	NO 2<br>YES 1	H2F) TRIED TO CHOKE OR BURN/SCALD YOUR PARTNER ON PURPOSE?	*IPV18 B*	NO 2<br>YES 1
H1G) THREATENED TO OR USED KNIFE, GUN OR ANY OTHER WEAPON TO HURT YOU?	*IPV19A*	NO 2<br>YES 1	H2G) THREATENED TO OR USED KNIFE, GUN OR ANY OTHER WEAPON TO HURT YOUR I PARTNER?	*IPV19 B*	NO 2<br>YES 1
H1H) USED FORCE TO HAVE SEXUAL INTERCOURSE WITH YOU WHEN YOU DID NOT WANT TO?	*IPV20A*	NO 2<br>YES 1	H2H) USED FORCE TO HAVE SEXUAL INTERCOURSE WITH YOUR PARTNER WHEN THEY DID NOT WANT TO?	*IPV20 B*	NO 2<br>YES 1
H1I) FORCED YOU TO DO ANY ACT THAT WAS SEXUAL AND THAT YOU DID NOT WANT TO DO?	*IPV21A*	NO 2<br>YES 1	H2I) FORCED YOUR PARTNER TO DO ANY ACT THAT WAS SEXUAL AND THAT THEY DID NOT WANT TO DO?	*IPV21 B*	NO 2<br>YES 1

"Now here are some others about you, please tell me if you disagree or agree with the statement. If you agree to the statement also tell me if you agree completely or only agree to some extent. If you disagree with the statement also tell me if you disagree to some extent or disagree completely with the statement."

SECTION 10: ROSENBURG SELF-ESTEEM SCALE

RS 1	I FEEL THAT I AM A PERSON OF WORTH, AT LEAST ON AN EQUAL BASIS WITH OTHERS.	Disagree completely .... 1<br>Disagree to some extent .... 2<br>Agree to some extent .... 3<br>Agree completely 4	
RS 2	ALL IN ALL, I HAVE DISAPPOINTED OTHERS AND MYSELF.	Disagree completely .... 1<br>Disagree to some extent .... 2<br>Agree to some extent .... 3<br>Agree completely 4	
RS 3	I AM ABLE TO DO THINGS AS WELL AS MOST PEOPLE.	Disagree completely .... 1<br>Disagree to some extent .... 2<br>Agree to some extent .... 3<br>Agree completely 4	
RS 4	I FEEL I DO NOT HAVE MUCH TO BE PROUD OF.	Disagree completely .... 1<br>Disagree to some extent .... 2<br>Agree to some extent .... 3<br>Agree completely 4	
RS 5	ON THE WHOLE, I AM SATISFIED WITH MYSELF.	Disagree completely .... 1<br>Disagree to some extent .... 2<br>Agree to some extent .... 3<br>Agree completely 4	
RS 6	I CERTAINLY FEEL COMPETENT AT TIMES.	Disagree completely .... 1<br>Disagree to some extent .... 2<br>Agree to some extent .... 3<br>Agree completely 4	
RS 7	AT TIMES, I THINK I AM NO GOOD AT ALL.	Disagree completely .... 1<br>Disagree to some extent .... 2<br>Agree to some extent .... 3<br>Agree completely 4	

"Over the **past two weeks** how often have you been bothered by the following problems?"

**SECTION 11: DEPRESSION**			
D 1	TROUBLE FALLING ASLEEP OR STAYING ASLEEP, OR SLEEPING TOO MUCH?	Not at all .... 0<br>Several days 1<br>More than half the days............................ 2<br>Nearly every day 3	
D2	FEELING TIRED OR HAVING LITTLE ENERGY?	Not at all .... 0<br>Several days .... 1<br>More than half the days .... 2<br>Nearly every day 3	

D3	POOR APPETITE OR OVER-EATING?	Not at all .... 0<br>Several days .... 1<br>More than half the days .... 2<br>Nearly every day 3	
D4	FEELING BAD ABOUT YOURSELF, OR THAT YOU ARE A FAILURE OR HAVE LET YOURSELF OR YOUR FAMILY DOWN?	Not at all .... 0<br>Several days .... 1<br>More than half the days .... 2<br>Nearly every day 3	
D5	TROUBLE CONCENTRATING ON THINGS, SUCH AS READING THE NEWSPAPER OR WATCHING TV?	Not at all .... 0<br>Several days .... 1<br>More than half the days .... 2<br>Nearly every day 3	
D6	MOVING OR SPEAKING SO SLOWLY THAT OTHER PEOPLE HAVE NOTICED? OR THE OPPOSITE- BEING SO FIDGETY AND RESTLESS THAT YOU HAVE BEEN MOVING AROUND A LOT MORE THAN USUAL?	Not at all .... 0<br>Several days .... 1<br>More than half the days .... 2<br>Nearly every day 3	
D7	THOUGHTS THAT YOU WOULD BE BETTER OFF DEAD OR OF HURTING YOURSELF IN SOME WAY?	Not at all .... 0<br>Several days .... 1<br>More than half the days .... 2<br>Nearly every day 3	
D8	LITTLE INTEREST OR PLEASURE IN DOING THINGS?	Not at all .... 0<br>Several days .... 1<br>More than half the days .... 2<br>Nearly every day 3	
D 9	FEELING DOWN, DEPRESSED, OR HOPELESS?	Not at all .... 0<br>Several days .... 1<br>More than half the days .... 2<br>Nearly every day 3	
D10	IF ALL ITEMS ARE 0, SKIP. IF ANY PROBLEMS WERE CHECKED OFF, ASK<br><br>HOW DIFFICULT HAVE THESE PROBLEMS MADE IT FOR YOU TO DO YOUR WORK, TAKE CARE OF THINGS AT HOME, OR GET ALONG WITH OTHER PEOPLE?	Extremely difficult .... 3<br>Very difficult .... 2<br>Somewhat difficult .... 1<br>Not difficult at all .... 0<br><br>N/A 99	

12A. During the past six months have did you have any alcoholic drinks?
Yes……………………………1
No………………………………2 If No Skip to Section 13

SECTION 12: RAPID ALCOHOL PROBLEM SCREENING

RAPS1	1. DURING THE LAST SIX MONTHS HAVE YOU HAD A FEELING OF GUILT OR REMORSE AFTER DRINKING?	Yes……………………………………………1<br>No……………………………………………..2	
RAPS 2	DURING THE LAST SIX MONTHS HAS A FRIEND OR FAMILY MEMBER EVER TOLD YOU ABOUT THINGS YOU SAID OR DID WHILE YOU WERE DRINKING THAT YOU COULD NOT REMEMBER?	Yes……………………………………………1<br>No……………………………………………..2	
RAPS 3	DURING THE LAST SIX MONTHS HAVE YOU FAILED TO DO WHAT WAS NORMALLY EXPECTED FROM YOU BECAUSE OF DRINKING?	Yes……………………………………………1<br>No……………………………………………..2	
RAPS 4	DO YOU SOMETIMES TAKE A DRINK WHEN YOU FIRST GET UP IN THE MORNING?	Yes……………………………………………1<br>No……………………………………………..2	
RAPS 5	DURING THE LAST SIX MONTHS HAVE YOU LOST FRIENDS OR GIRLFRIENDS OR BOYFRIENDS BECAUSE OF DRINKING?	Yes……………………………………………1<br>No……………………………………………..2	

SECTION 13: NETWORK ELICITATION AND SOCIAL STRUCTURE
I'd like you to name 12-15 people who you know and whom they know you.. These should be people that you have had a conversation with, phone call or email in the past 30 days. They can be sexual partners, friends, community members or community leaders, religious leaders or ministers, family members etc. You should not include people involved in providing you with HIV care or support, such as doctors, nurses, and counselors. Please name adults age 18 or older only-- do not give me names of children under 18. Please give us the person's first name and first two initials only of their last name. We also need a nickname that you use for this person. We do NOT want to know their last names, so that we can protect their confidentiality. Just make sure that you will be able to remember them as we move forward with other questions.

**1**
**2**
**3**
**4**
**5**
**6**
**7**
**8**
**9**
**10**
**11**
**12**
**Are there any other people that you would like to name?**
**Yes…….1 No…….2 If No skip to NM1**
**13**
**14**
**15**

1. NETWORK MEMBERS DEMOGRAPHICS AND RELATIONSHIP TO CHANGE AGENT

I need to ask you some questions about the people you named before. Let's just go down the list of initials for each question and you tell me the information to the best of your knowledge. It's fine to estimate or give an educated guess if you're not sure [Will be repeated for each of the 12-15 Network Members]	
**QUESTIONS ON NETWORK DEMORAPHICS**	**RESPONSES**
**NM 1. What is [initials] age?**	Age in years
**NM 2. What is [initials] gender?**	Female = 0, Male = 1
**NM 3. What is [initials] relationship to you?**	Family member=1, Friend=2, Sexual partner=3, Church member=4, , Support group member=8, Co-worker=9;<br>Other=10 specify: ___________________
**NM 4. What other type of relationship does [initials] have to you?**	Explanation of relationship
**NM 5. IF SEXUAL PARTNER, ASK: Are you using condoms with [initials] in the past month?**	Yes, all of the time= 1, Yes, some of the time= 2, No = 3
**NM 6. IF FAMILY MEMBER, ASK: What type of family member is [initials]?**	Spouse=0,grandmother=1, grandfather=2, mother=3, father=4, brother=5, sister=6, aunt=7, uncle=8, cousin=9, daughter=10 (18 years or older), son=11 (18 years or older),<br>other=12, specify: ______________________________
**NM 7. What is [initials] HIV status?**	Negative=1, Positive=2, Unknown=88
**NM 8. If positive, is [initials] receiving HIV care?**	1=Yes; 2= No, DK/not sure = 88
**NM 9. If positive, is [initials] on HIV antiretroviral treatment?**	1=Yes; 2= No, DK/not sure = 88
**NM 10. Does [initials] know that you are HIV-positive?**	1=Yes; 2= No, DK/Not sure=88
**NM 11. How much do you see or talk to [initials] in a typical month?**	Never=1, One time a month=2, One time a week=3, Several times a week=4, About every day=5
**NM 12. How close are you to [initials]?**	Not at all close=1, somewhat close=2, very close=3
**NM 13. How much do you trust [initials]?**	Not at all=1, somewhat=2 very much=3
**NM 14. How often have you had arguments with, or gotten upset with [initials]?**	Never=1, sometimes=2, often=3
**NM 15. How much do you ask [initials] for advice or information when you have a problem?**	Never=1, sometimes=2, often=3
**NM 16. How often has [initials] provided you with food, money, clothes or a place to stay when needed?**	never=1, sometimes=2, often=3
**NM 17. How often do you share your thoughts and feelings with [initials]?**	Never=1, sometimes=2, often=3
**NM 18. How often will [initials] comfort you?**	Never=1, sometimes=2, often=3
**NM 19. Would [initials] loan you money?**	1=Yes; 2= No, DK/Not sure=88

**NM 20. Would [initials] offer you a ride (on a bicycle, car etc)?**	1=Yes; 2= No DK/Not sure=88
**NM 21. I feel that [initials] show me enough consideration.**	Not at all=1, somewhat=2, very much=3
**NM 22. [Initials] treats me fairly and justly.**	Never=1, sometimes=2, often=3
**NM 23. I feel that [initials] can be counted on to help me.**	Not at all=1, somewhat=2, very much=3
**NM 24. How often does [initials] talk to you about HIV?**	never=1, sometimes=2, often=3

COMMUNICATING HIV ATTITUDES, BELIEFS, BEHAVIORS WITH NETWORK MEMBERS

I would like to ask you a few questions about discussions related to HIV that you may or may not have had with any of these people in your life. Let's just go down the list of initials of each person, and I will ask whether you have had certain types of discussions with them over the past 3 months, and if so, how often, and whether to the best of your knowledge those discussions had any impact on that person's behavior. **[Questions will be repeated for each of 20 Network Members]**	
**QUESTIONS ON CONTENT OF HIV COMMUNICATION WITH NETWORK MEMBERS**	**RESPONSES**
C 1. How often do you talk to [initials] about abstinence or using condoms when having sex?	never=1, sometimes=2, often=35
**Ask the following if the social network member's HIV status is negative or unknown**	
C 2How often do you talk to [initials] about being tested for HIV?	never=1, sometimes=2, often=3
**Ask the following if the social network member is HIV+**	
C 3. How often do you talk to [initials] about protecting others from HIV?	never=1, sometimes=2, often=3
**If HIV+ and not in care, ask the following:**	
C 4. How often do you talk to [initials] about seeking medical care from an HIV clinic?	never=1, sometimes=2, often=3
**If HIV+, pregnant, and not on ART, ask the following:**	
C 5. How often do you talk to [initials] about various strategies for prevention of mother to child transmission?	never=1, sometimes=2, often=3

ASSESSING APPROPRIATENESS FOR INTERVIEWING

I would like to ask you a few questions about your perception of this person. **[Questions will be repeated for each of 20 Network Members]**	
**QUESTIONS**	**RESPONSES**
C 6. Do you think [initials] might be at risk for HIV. The definition of at risk includes people who themselves, or whose partners might be having unsafe sex, have more than 1 recent sexual partner, inject drugs, don't use condoms with casual partners, or may be experiencing violence in their relationships.	1=Yes; 2= No
C 7. Do you feel comfortable inviting [initials] to be in a health survey?	1=Yes; 2= No

"This is the end of the questionnaire. Thank you very much for your time and for your willingness to answer the questions."
