# Supplementary material for: Agents of change: Comparing HIV-related risk behavior of people attending ART clinics in Dar es Salaam with members of their social networks
Source: PLoS One. 2020 Sep 4;15(9):e0238240. doi: 10.1371/journal.pone.0238240 (PMC7473570; doi:10.1371/journal.pone.0238240)
Supplement: S2 Appendix — (PDF) [file pone.0238240.s002.pdf]

Appendix C.2 Interviews with Network Members: Baseline Questionnaire

CAP MC PHE PROTOCOL

INTERVIEWS WITH NETWORK MEMBERS: BASELINE QUESTIONNAIRE

ID number ☐☐☐☐☐

First 2 initials of last name: ☐☐

Change Agent ID number: ☐☐☐

Date of assessment: ☐☐/☐☐/☐☐☐☐ (dd/mm/yyyy)

SCREENING QUESTION		
	THANK YOU FOR COMING TO LEARN MORE ABOUT THIS RESEARCH PROJECT. BEFORE WE START DO YOU AGREE TO INFORM ME ABOUT HOW YOU LEARNT ABOUT THIS STUDY?<br><br>**[IF YES TICK REGISTER TO INDICATE ACCEPTS TO DISCUSS WHO REFERRED HIM/HER TO THE AKC SITE (SCREENING)**<br><br>WHO REFERRED YOU TO THIS STUDY?<br><br>(NOTE-CHECK THAT THIS NAME MATCHES THE NAME FOR LISTED FOR THE ID NUMBER ON THE LETTER HANDED TO YOU BY THE PARTICIPANT. IF THE NAME DOES NOT TALLY, THANK THE PERSON AND EXPLAIN THEY ARE NOT ELIGIBLE) TALLY THIS PERSON AS NOT ELIGIBLE	NAME:<br><br>……………………………………

Let me read for you what the research project is about, the risks and benefits of your participation and people you will be able to contact for more information about the research project if you agree to participate. Please do not hesitate to ask any questions so that you understand in what ways you will be participating, should you accept to do so. **[read NWM consent form and get signed consent]**

Thank you for accepting to be an important part of this research project to improve health in the community. Your name was given to us by one of those participating in this training program, [xxx], identified you as part of his/her network of family and friends.

"May I conduct the interview now?" *If permission is given, begin the interview.*

"I am going to start by asking you some background information about yourself. I am then going to ask you about your relationship with the person who told you about this study, [XX]."

SECTION 1: BASELINE			
B 1	WHAT IS YOUR AGE?	Years .... __ __	
B 2	WHAT IS YOUR DATE OF BIRTH?	Day __ __ Month __ __ Year __ __ __ __	

B 3	WHAT IS YOUR GENDER?	Male ....1<br>Female..2	
B 4.1	IF FEMALE, ASK: ARE YOU CURRENTLY PREGNANT?	No .... 2<br>Yes .... 1<br><br>Don't know .... 88<br>Refused 77	IF 7,8<br>◆B5
B4.2	IF CURRENTLY PREGNANT WHEN WAS THE FIRST DAY OF YOUR LAST PERIOD	Day __ __ Month __ __ Year __ __ __ __	
B5	WHAT IS YOUR CURRENT MARITAL STATUS?	Married ....1<br>Cohabiting ....2<br>Single...3<br>Divorced/Separated...................................4<br>Widowed..…… 5	
B6	HOW MANY YEARS OF SCHOOL HAVE YOU COMPLETED? [COMPLETE ALL CATEGORIES WITH NUMBER OF YEARS OF SCHOOLING; IF THE PERSON HAD NO EDUCATION, INDICATE '0' IN THE TOTAL LINE FOR THIS ITEM.]	Primary Education .... _____<br><br>Secondary Education .... _____<br>Certificate/Diploma/ Advanced diploma or Other non-degree professional training .... _____<br>University/ Degree level professional training_____	
B7	WHAT IS YOUR CURRENT EMPLOYMENT STATUS? [SELECT PRIMARY OCCUPATIONAL STATUS.]	Employed .... 1<br>Self-employed .... 2<br>Housewife/house husband .... 3<br>Unemployed .... 4<br>Student ……………………………………...5	
B8	IN YOUR CURRENT RESIDENCE, HOW MANY ROOMS DO YOU HAVE FOR THE USE OF YOU AND YOUR FAMILY?	Rooms .... __ __	

B9	IN YOUR HOUSE, HOW MANY ROOMS ARE USED FOR SLEEPING?	Rooms .... __ __	
B10	HOW MANY PEOPLE, INCLUDING YOURSELF, USUALLY SLEEP IN YOUR HOUSEHOLD?	People .... __ __	
B11	WHAT IS THE MAIN SOURCE OF ENERGY FOR LIGHTING IN YOUR HOUSEHOLD?	Solar .... 1<br>Gas .... 2<br>Paraffin/Hurricane lamp............................. 3<br>Paraffin/Pressure lamp .... 4<br>Paraffin wick lamp .... 5<br>Electricity ……………………………………6<br>Other .... 7<br>*(specify)* ___________________________	
B12.1	WHAT IS THE MAIN MATERIAL OF THE FLOOR IN YOUR HOUSEHOLD?	Earth and or sand .... 1<br>Dung .... 2<br>Rudimentary floor .... 3<br>Wooden floor .... 4<br>Vinyl or asphalt strips .... 5<br>Cement screed .... 6<br>Tiles .... 7<br>Other .... 8<br>*(specify)* ___________________________	
B12.2	WHAT IS THE MAIN MATERIAL OF THE ROOF IN YOUR HOUSEHOLD?	Grass/Leaves/ .... 1<br>Iron sheets .... 2<br>Tiles .... 3<br>Concrete .... 4<br>Other .... 5<br>*(specify)* ___________________________	

B12.3	WHAT IS THE MAIN MATERIAL OF THE WALL IN YOUR HOUSEHOLD?	Poles and mud... 1<br>Sundried bricks.. 2<br>Baked bricks... 3<br>Timber .... 4<br>Cement bricks... 5<br>Stones .... 6<br>Other .... 7<br>*(specify)* ________________________	
B13	WHAT IS THE MAIN SOURCE OF DRINKING WATER FOR YOUR HOUSEHOLD?	Piped into dwelling/yard/plot .... 1<br>Public/Neighbour's tap .... 2<br>Open well in dwelling/yard/plot .... 3<br>Open public/neighbour's well .... 4<br>Protected well in dwelling/yard/plot .... 5<br>Protected public/neighbour well .... 6<br>Spring .... 7<br>River, stream .... 8<br>Pond/Lake/Dam... 9<br>Tanker truck .... 10<br>Water vendor .... 11<br>Other .... 12<br>*(specify)* ________________________	
B14	HOW OFTEN IN THE LAST 6 MONTHS DID YOUR HOUSEHOLD HAVE PROBLEMS IN SATISFYING THEIR FOOD NEEDS?	Never…………………………………………1<br>Seldom ……………………………………...2<br>Sometimes……………………………………3<br>Often…………………………………………..4<br>Always…………………………………………5	
B15	WHEN YOU ARE IN YOUR HOUSEHOLD, WHAT KIND OF TOILET FACILITY DO YOU USUALLY USE?	Flush toilet used by household/family only 1<br>Pit latrine used by household/family only .. 2<br>Flush toilet shared with other tenants........ 3<br>Pit Latrine shared with other tenants......... 4<br>Flush toilet shared with a neighbor in different house .... 5<br>No facility/Bush/Field 6	
B16	HOW MANY CHILDREN (UNDER 18 YEARS OF AGE) ARE CURRENTLY LIVING IN YOUR HOUSEHOLD?	Children .... __ __	

B 17	WHAT IS YOUR RELATIONSHIP TO THE PERSON WHO REFERRED YOU TO THIS SURVEY LET US CALL HIM/HER XX?	Family Member .... 0<br>Friend .... 1<br>Sex/ Relationship partner .... 2<br>Church member .... 3<br>Support group member .... 4<br>Other .... 5<br>*specify:*<br>……………………………………………	IF<br>1-8<br>SKIP<br>TO⇨<br>Q.B21
B 18	[IF FAMILY MEMBER, ASK:] WHAT TYPE OF FAMILY MEMBER ARE YOU TO [XX]? ARE YOU A….	Spouse……………………………………..0<br>Grandmother .... 1<br>Grandfather .... 2<br>Mother .... 3<br>Father .... 4<br>Brother .... 5<br>Sister .... 6<br>Aunt .... 7<br>Uncle .... 8<br>Cousin .... 9<br>Adult daughter…………………………...10<br>Adult son……………………………………11<br>Other ……………………………………………12<br>*specify:*<br>…………………………………….	
B 19	HOW OFTEN DO YOU MEET AND TALK TO [XX]?	About every day .... 5<br>About several times a week .... 4<br>About once a week .... 3<br>About several times a month………………2<br>About once a month .... 1<br>Less than once a month 0	
B 20	HOW MUCH DO YOU APPRECIATE AND VALUE [XX]?	Very much .... 4<br>Quite a lot .... 3<br>Some .... 2<br>A little .... 1<br>Not at all 0	
B 21	HOW MUCH DO YOU LISTEN TO ADVICE FROM [XX]?	Very much .... 4<br>Quite a lot .... 3<br>Some .... 2<br>A little .... 1<br>Not at all 0	

B 22	IN THE PAST 6 MONTHS, HOW OFTEN DO YOU AND [XX] ENJOY GOOD CONVERSATIONS /TIMES TOGETHER?	About every day .... 5<br>About several times a week .... 4<br>About once a week.................................... 3<br>About several times a month……………..2<br>About once a month .... 1<br>Less than once a month 0	
B 23	HOW EASY OR DIFFICULT IS IT FOR YOU TO ASK [XX] FOR ADVICE OR INFORMATION?	Very easy.. 3<br>Easy... 2<br>difficult .... 1<br>Very difficult 0	
B 24	HOW EASY OR DIFFICULT IS IT FOR YOU TO ASK [XX] FOR ANY OTHER ASSISTANCE WHEN YOU NEEDED IT?	Very easy.. 3<br>Easy... 2<br>difficult .... 1<br>Very difficult 0	
B 25	HOW EASY OR DIFFICULT IS IT FOR YOU TO GIVE ASSISTANCE TO OTHERS WHEN THEY NEED IT?	Very easy.. 3<br>Easy... 2<br>difficult .... 1<br>Very difficult 0	
B26.1	HOW OFTEN DO YOU DISCUSS QUESTIONS RELATED TO MALARIA WITH OTHERS?	Often ………………………………………..3<br>Sometimes …………………………………2<br>Seldom ………………………………………1<br>Never …………………………………………0	
B26.2	HOW OFTEN DO YOU DISCUSS QUESTIONS RELATED TO HIV WITH OTHERS?	Often ………………………………………..3<br>Sometimes …………………………………2<br>Seldom ………………………………………1<br>Never …………………………………………0	
B27.1	HOW OFTEN DO YOU AND [XX] DISCUSS QUESTIONS RELATED TO MALARIA?	Often ………………………………………..3<br>Sometimes …………………………………2<br>Seldom ………………………………………1<br>Never …………………………………………0	
B 27.2	HOW OFTEN DO YOU AND [XX] DISCUSS QUESTIONS RELATED TO HIV?	Often ………………………………………..3<br>Sometimes …………………………………2<br>Seldom ………………………………………1<br>Never …………………………………………0	

SECTION 2: ROSENBURG SELF-ESTEEM SCALE			
R 1	I FEEL THAT I AM A PERSON OF WORTH, AT LEAST ON AN EQUAL BASIS WITH OTHERS.	Disagree completely .... 1<br>Disagree to some extent .... 2<br>Agree to some extent .... 3<br>Agree completely 4	
R 2	ALL IN ALL, I HAVE DISAPPOINTED OTHERS AND MYSELF.	Disagree completely .... 4<br>Disagree to some extent .... 3<br>Agree to some extent .... 2<br>Agree completely 1	
R 3	I AM ABLE TO DO THINGS AS WELL AS MOST PEOPLE.	Disagree completely .... 1<br>Disagree to some extent .... 2<br>Agree to some extent .... 3<br>Agree completely 4	
R 4	I FEEL I DO NOT HAVE MUCH TO BE PROUD OF.	Disagree completely .... 4<br>Disagree to some extent .... 3<br>Agree to some extent .... 2<br>Agree completely 1	
R 5	ON THE WHOLE, I AM SATISFIED WITH MYSELF.	Disagree completely .... 1<br>Disagree to some extent .... 2<br>Agree to some extent .... 3<br>Agree completely 4	
R 6	I CERTAINLY FEEL COMPETENT AT TIMES.	Disagree completely .... 1<br>Disagree to some extent .... 2<br>Agree to some extent .... 3<br>Agree completely 4	
R 7	AT TIMES, I THINK I AM NO GOOD AT ALL.	Disagree completely .... 4<br>Disagree to some extent .... 3<br>Agree to some extent .... 2<br>Agree completely 1	

SECTION 3: GENERAL QUESTIONS ABOUT HEALTH AND ACCESSING HEALTH CARE FOR COMMON AND SERIOUS MEDICAL ILLNESSES			
B 28	HAS ANYONE IN YOUR HOUSEHOLD RECEIVED MEDICAL SUPPLIES FOR AN ILLNESS IN THE PAST THREE MONTHS? (IF YES ASK Q 27)	No .... 2<br>Yes .... 1<br><br>Do not know... 88	IF 2 SKIP TO⇨ Q.28
B 29	IF YES, FOR WHAT CONDITIONS? (PROBE: ANY OTHER CONDITIONS?)	Malaria<br>No .... 2<br>Yes .... 1<br><br>Anaemia<br>No .... 2<br>Yes .... 1<br><br>Diabetes<br>No .... 2<br>Yes .... 1<br><br>Tuberculosis<br>No .... 2<br>Yes .... 1<br><br>Hypertension<br>No .... 2<br>Yes .... 1<br><br>Mental disorder<br>No .... 2<br>Yes .... 1<br><br>HIV/AIDS<br>No .... 2<br>Yes .... 1<br><br>Other<br>No .... 2<br>Yes 1	

B 30	HOW IMPORTANT DO YOU THINK THAT IT IS TO BE TESTED PROMPTLY FOR MALARIA IF YOU HAVE FEVER?	Very important .... 4<br>Important .... 3<br>Somewhat important................................. 2<br>A little important.. 1<br>Not at all important 0	
B 31	HOW IMPORTANT DO YOU THINK THAT IT IS TO BE TESTED FOR HIV?	Very important .... 4<br>Important .... 3<br>Somewhat important................................. 2<br>A little important.. 1<br>Not at all important 0	
B 32	HAVE YOU EVER BEEN TESTED FOR HIV?	No .... 2<br>Yes .... 1<br>Refused to respond 77	IF 1 or 7 SKIP TO⇨ Q.AK1
B33	WHY WERE YOU TESTED?	I was sick……………………………………6<br>I was having sex with a risky partner………..5<br>My friend influenced me……………………..4<br>I was offered a test health care worker………3<br>I saw an advert (tv, radio, billboard or newspaper) ..2<br>Other……………………………………………..1<br>Please specify ________________________	
B 34	WHAT WAS THE DATE, (MONTH AND YEAR), OF YOUR LAST HIV TEST?	Month .... __ __<br>Year .... __ __ __ __<br>Don't know... 88<br>Refused to answer.................................. 77	
B 35	WHAT IS YOUR HIV STATUS?	Negative .... 2<br>Positive .... 1<br>Have not been tested………………………3<br>Refused to respond 77	IF 2 OR 7 SKIP TO⇨ AK1
B 36	IF HIV+VE FOR Q.35, ASK: HAVE YOU VISITED A HIV TREATMENT CLINIC?	No .... 2<br>Yes 1	

B 37	IF YES FOR Q. 36, ASK: HAVE YOU STARTED RECEIVING HIV ANTIRETROVIRAL TREATMENT?	No .... 2<br>Yes .... 1<br>N/A........99	
B38	APART FROM CLINIC STAFF, HAVE YOU DISCLOSED YOUR HIV STATUS TO?	Sexual partner/spouse .... yes=1/no=2<br>Family Member .... yes=1/no=2<br>Friend .... yes=1/no=2<br>Church member .... yes=1/no=2<br>Community leader .... yes=1/no=2<br>Social services provider .... yes=1/no=2<br>Support group member .... yes=1/no=2<br>Other .... yes=1/no=2<br>*specify:*........	B38.1<br>B38.2<br>B38.3<br>B38.4<br>B38.5<br>B38.6<br>B38.7<br>B38.8
B 39	HAVE ANY OF THE FOLLOWING DISCLOSED THEIR HIV STATUS TO YOU?	Sexual partner/spouse .... yes=1/no=2<br>Family Member .... yes=1/no=2<br>Friend .... yes=1/no=2<br>Other........ yes=1/no=2<br>*specify*	B39.1<br>B39.2<br>B39.3<br>B39.4

"I would like to ask you some questions about your knowledge of HIV/AIDS."

**SECTION 4: HEALTH PROMOTION KNOWLEDGE**			
AK 1	CAN PEOPLE REDUCE THEIR CHANCE OF GETTING THE AIDS VIRUS BY HAVING JUST ONE UNINFECTED SEX PARTNER WHO HAS NO OTHER SEX PARTNERS?	No ....2<br>Yes........1<br>Don't know........88	
AK 2	CAN PEOPLE GET THE AIDS VIRUS FROM MOSQUITO BITES?	No ....2<br>Yes........1<br>Don't know........88	
AK 3	CAN PEOPLE REDUCE THEIR CHANCE OF GETTING THE AIDS VIRUS BY USING A CONDOM EVERY TIME THEY HAVE SEX?	No ....2<br>Yes........1<br>Don't know........88	

AK 4	CAN PEOPLE GET THE AIDS VIRUS BY SHARING FOOD WITH A PERSON WHO HAS AIDS?	No ....2<br>Yes..1<br>Don't know 88	
AK 5	CAN PEOPLE REDUCE THEIR CHANCE OF GETTING THE AIDS VIRUS BY NOT HAVING SEXUAL INTERCOURSE AT ALL?	No ....2<br>Yes..1<br>Don't know 88	
AK 6	CAN PEOPLE GET THE AIDS VIRUS BECAUSE OF WITCHCRAFT OR OTHER SUPERNATURAL MEANS?	No ....2<br>Yes..1<br>Don't know 88	
AK 7	IS IT POSSIBLE FOR A HEALTHY-LOOKING PERSON TO HAVE THE AIDS VIRUS?	No ....2<br>Yes..1<br>Don't know 88	
AK 8	IF A MAN HAS THE VIRUS THAT CAUSES AIDS DOES HIS SEXUAL PARTNER ALWAYS, ALMOST ALWAYS, OR ONLY SOMETIMES HAVE THE AIDS VIRUS?	Always ....1<br>Almost always..2<br>Sometimes...3<br>Don't know 88	
AK 9	IF A WOMAN HAS THE VIRUS THAT CAUSES AIDS DOES HER SEXUAL PARTNER ALWAYS, ALMOST ALWAYS, OR ONLY SOMETIMES HAVE THE AIDS VIRUS?	Always ....1<br>Almost always..2<br>Sometimes...3<br>Don't know 88	
AK 10	CAN THE VIRUS THAT CAUSES AIDS TRANSMITTED FROM A MOTHER TO HER BABY:	No ....2<br>Yes..1<br>Don't know 88	

AK 10.1	DURING PREGNANCY?	No .... 2<br>Yes .... 1<br>Don't know 88	

AK 10.2	DURING DELIVERY?<br><br>IF NO SKIP TO HK 11	No .... 2<br>Yes .... 1<br>Don't know 88	
AK 10.3	BY BREASTFEEDING?	No .... 2<br>Yes .... 1<br>Don't know 88	
AK 11.1	ARE THERE SPECIAL DRUGS THAT A DOCTOR OR A NURSE CAN GIVE TO A WOMAN INFECTED WITH THE AIDS VIRUS TO REDUCE THE RISK OF TRANSMISSION TO THE BABY?	No .... 2<br>Yes .... 1<br>Don't know 88	
AK 11.2	HAVE YOU HEARD ABOUT SPECIAL ANTIRETROVIRAL DRUGS (USE LOCAL NAME) THAT PEOPLE INFECTED WITH THE VIRUS CAN GET FROM A DOCTOR OR A NURSE TO HELP THEM LIVE LONGER?	No .... 2<br>Yes 1	

AK 12.1	WHAT ARE THE MAIN CHANNELS OF COMMUNICATION FROM WHICH YOU RECEIVE INFORMATION AND EDUCATION ABOUT MALARIA?<br><br>[SELECT ALL THAT APPLY.]<br><br>PROBE: ANY OTHER CHANNELS?	Radio<br>No .... 2<br>Yes.. 1<br><br>Television<br>No .... 2<br>Yes.. 1<br><br>Film<br>No .... 2<br>Yes.. 1<br><br>Drama<br>No .... 2<br>Yes.. 1<br><br>Newspapers/magazines<br>No .... 2<br>Yes.. 1<br><br>Brochures<br>No .... 2<br>Yes.. 1<br><br>Posters<br>No .... 2<br>Yes.. 1<br><br>Billboards<br>No .... 2<br>Yes.. 1<br><br>Community notices<br>No .... 2<br>Yes.. 1<br><br>Family<br>No .... 2<br>Yes.. 1<br><br>Friends<br>No .... 2<br>Yes.. 1<br><br>Peers<br>No .... 2<br>Yes.. 1<br><br>Health workers<br>No 2	

(AK 12.1)	(CONTD) WHAT ARE THE MAIN CHANNELS OF COMMUNICATION FROM WHICH YOU RECEIVE INFORMATION AND EDUCATION ABOUT MALARIA?<br><br>[SELECT ALL THAT APPLY.]<br><br>PROBE: ANY OTHER CHANNELS?	Traditional leaders<br>No ....2<br>Yes..1<br><br>Religious leaders<br>No ....2<br>Yes..1<br><br>Internet<br>No ....2<br>Yes..1<br>Other<br>No ....2<br>Yes..1<br>(specify) _____________<br>NONE...0<br>Refused to answer...................................77	

AK 12.2	WHAT ARE THE MAIN CHANNELS OF COMMUNICATION FROM WHICH YOU RECEIVE AIDS INFORMATION AND EDUCATION?<br><br>[SELECT ALL THAT APPLY.]<br><br>PROBE: ANY OTHER CHANNELS?	Radio<br>No .... 2<br>Yes .... 1<br><br>Television<br>No .... 2<br>Yes .... 1<br><br>Film<br>No .... 2<br>Yes .... 1<br><br>Drama<br>No .... 2<br>Yes .... 1<br><br>Newspapers/magazines<br>No .... 2<br>Yes .... 1<br><br>Brochures<br>No .... 2<br>Yes .... 1<br><br>Posters<br>No .... 2<br>Yes .... 1<br><br>Billboards<br>No .... 2<br>Yes .... 1<br><br>Community notices<br>No .... 2<br>Yes .... 1<br><br>Family<br>No .... 2<br>Yes .... 1<br><br>Friends<br>No .... 2<br>Yes .... 1<br><br>Peers<br>No .... 2<br>Yes 1	

(AK 12.2)	{CONTD] WHAT ARE THE MAIN CHANNELS OF COMMUNICATION FROM WHICH YOU RECEIVE AIDS INFORMATION AND EDUCATION?<br><br>(PROBE: ANY OTHER CHANNELS?)	Traditional leaders<br>No ....2<br>Yes..1<br><br>Religious leaders<br>No ....2<br>Yes..1<br><br>Internet<br>No ....2<br>Yes..1<br><br>Other<br>No ....2<br>Yes..1<br>(specify) _____________<br><br>NONE<br><br>Refused to answer…………………77	

"I would like to ask you a few questions about discussions related to health that you may or may not have had with [XX] **over the past 3 months**. I will also ask whether or not you have engaged in specific health protective behaviors, and whether or not the discussions you had with [XX] had any impact on these behaviors."

SECTION 5: HIV ATTITUDES, BELIEFS, BEHAVIORS			
BB 1	HOW OFTEN HAS [XX] TALKED WITH YOU ABOUT HOW HAVING FEWER SEXUAL PARTNERS CAN LIMIT THE SPREAD OF HIV?	Often …… …………………………………..3<br>Sometimes …………………………………..2<br>Seldom ………………………………………1<br>Never.. ………………………………………0	
BB 2	TO WHAT EXTENT HAVE THESE DISCUSSIONS INFLUENCED THE WAY YOU ACT WITH SEXUAL PARTNERS?	Large extent .... 3<br>Moderate extent .... 2<br>Rarely .... 1<br>None... 0<br>N/A, not sexually active 99	

BB 3	HOW OFTEN HAS [XX] TALKED WITH YOU ABOUT THE BENEFIT OF USING A CONDOM WHEN HAVING SEX?	Often ...3<br>Sometimes ...2<br>Seldom ...1<br>Never ...0	
BB 4	HOW OFTEN DO YOU USE CONDOMS WITH CASUAL PARTNERS?	Often ...3<br>Sometimes ...2<br>Seldom ...1<br>Never ...0	
BB 5	HOW OFTEN HAS [XX] TALKED TO YOU ABOUT HOW IT IS POSSIBLE TO PROTECT YOURSELVES FROM HIV	Often ...3<br>Sometimes ...2<br>Seldom ...1<br>Never ...0	
BB 6	HOW OFTEN HAS [XX] ENCOURAGED YOU TO GET TESTED FOR HIV?	Often ...3<br>Sometimes ...2<br>Seldom ...1<br>Never ...0	
	**IF THE SOCIAL NETWORK MEMBER IS HIV+, ASK THE FOLLOWING (IF SECTION 1 B35 IS HIV +VE) :**		
BB 7	HOW OFTEN HAS [XX]TALKED TO YOU ABOUT HOW IT IS POSSIBLE FOR YOU TO PROTECT OTHERS FROM GETTING HIV?	Often ...3<br>Sometimes ...2<br>Seldom ...1<br>Never ...0<br>N/A ... 99	
BB 8	ARE YOU CURRENTLY ENROLLED IN CARE FOR HIV?	No ... 2<br>Yes ... 1<br><br>N/A ... 99	
	**IF THE SOCIAL NETWORK MEMBER IS HIV+ AND NOT IN CARE, ASK THE FOLLOWING:**		

BB 9	HOW OFTEN HAS [XX] ENCOURAGED YOU TO SEEK CARE AND TREATMENT FOR HIV?	Often ……………………………………..3<br>Sometimes ………………………………..2<br>Seldom ……………………………………1<br>Never ……………………………………0<br>N/A 99	
BB 10	HAVE YOU RECEIVED HIV MEDICAL CARE OR SEEN AN HIV DOCTOR SINCE YOU HAD THOSE DISCUSSIONS?	No .... 2<br>Yes .... 1<br><br>N/A 99	
	**IF THE SOCIAL NETWORK MEMBER IS HIV+, PREGNANT, AND NOT IN CARE, ASK THE FOLLOWING:**		
BB 11	HOW OFTEN HAVE YOU TALKED WITH [XX] ABOUT BEING ON HIV MEDICATION TO PREVENT TRANSMITTING HIV TO THE BABY?	Often ……………………………………..3<br>Sometimes ………………………………..2<br>Seldom ……………………………………1<br>Never ……………………………………0	
BB 12	HAS THE DISCUSSION ENCOURAGED YOU TO START ART TO PREVENT HIV TRANSMISSION TO THE BABY?	No .... 2<br>Yes .... 1<br><br>N/A 99	

"Now here are some others about you, please tell me if you disagree or agree with the statement. If you agree to the statement also tell me if you agree completely or only agree to some extent. If you disagree with the statement also tell me if you disagree to some extent or disagree completely with the statement."

"Please indicate how confident you are that you could perform each of the following tasks."

SECTION 6: THE GENERAL SELF-EFFICACY SCALE

G 1	IF SOMEONE OPPOSES ME, I CAN FIND THE MEANS AND WAYS TO GET WHAT I WANT.	Not at all true .... 1<br>Hardly true .... 2<br>Moderately true .... 3<br>Exactly true 4	
G 2	I AM CONFIDENT THAT I COULD DEAL EFFICIENTLY WITH UNEXPECTED EVENTS.	Not at all true .... 1<br>Hardly true .... 2<br>Moderately true .... 3<br>Exactly true 4	
G 3	THANKS TO MY RESOURCEFULNESS, I KNOW HOW TO HANDLE UNFORESEEN SITUATIONS.	Not at all true .... 1<br>Hardly true .... 2<br>Moderately true .... 3<br>Exactly true 4	
G 4	WHEN I AM CONFRONTED WITH A PROBLEM, I CAN USUALLY FIND SEVERAL SOLUTIONS.	Not at all true .... 1<br>Hardly true .... 2<br>Moderately true .... 3<br>Exactly true 4	
G 5	IF I AM IN TROUBLE, I CAN USUALLY THINK OF A SOLUTION.	Not at all true .... 1<br>Hardly true .... 2<br>Moderately true .... 3<br>Exactly true 4	

Now I am going to ask you some personal questions about your life. This information will be kept confidential. If we should come to a question that you do not want to answer, just let me know and we will go on to the next question."

**SECTION 7: SEXUAL RELATIONSHIPS**					
S 1.		AT WHAT AGE DID YOU FIRST HAVE SEXUAL INTERCOURSE?			YEARS _ _

S<br>2		HAVE YOU HAD SEXUAL INTERCOURSE IN THE LAST 6 MONTHS?			NO 2<br>YES 1<br><br>IF NO⇨ SKIP TO
S<br>3.		HOW MANY SEXUAL PARTNERS HAVE YOU HAD IN THE LAST 6 MONTHS?			NUMBER OF SEXU<br>__

S4.	4. WHEN DID YOU FIRST START A RELATIONSHIP WITH THIS PARTNER?	5. WHAT IS THIS PARTNER'S RELATIONSHIP TO YOU [READ ALL RESPONSE CHOICES]?	6. HOW OFTEN HAVE YOU HAD SEX WITH THIS PERSON IN THE PAST 6 MONTHS?	7. HOW OFTEN DID YOU USE CONDOMS WITH THIS PERSON IN THE PAST 6 MONTHS?	8. HOW MANY TIMES DID YOU HAVE SEX WITH THIS PARTNER IN THE PAST 30 DAYS?	9 HOW MANY TIMES DID YOU USE A CONDOM DURING SEX WITH THIS PARTNER IN THE PAST 30 DAYS?		10. WHY DID YOU NOT USE A CONDOM WITH THIS PARTNER AT ALL TIMES?	11. WHEN DID YOU FIRST HAVE SEX WITH THIS PARTNE R?	12. WHEN DID YOU LAST HAVE SEX WITH THIS PARTNER?	13. DO YOU PLAN TO HAVE SEX WITH THIS PERSO N AGAIN ?	14 .WHAT IS THE HIV STATU S OF YOUR PARTN ER?	15. HAVE YOU SHARED YOUR HIV STATUS WITH THIS PARTNER	16. HAVE YOU HA SEX WITH ANY OTHER PART IN THE LAST 6 MONTHS
NP1	Year _ _<br>Month _ _<br>Day _ _<br><br>Refused. 77<br>N/A … 99	Spouse ………..1<br>Cohabitating partner…… 2<br>Non-cohabitating regular partner…….3<br>Casual partner (friend/other).4<br>Commercial sex worker/client……...5<br>Other (specify)….6<br>*(specify)* ________<br><br>Refused.……77<br>N/A……………99	Only once ….1<br>More than once but less than monthly.........2<br>About once per month………3<br>More than once per month but less than weekly……...4<br>Weekly……..5<br><br>Refused…77	Never ……...1<br>Some of the time ………..2<br>All of the time ..... ………..…3<br>Refused …77	Times _ _<br><br>If 00⇨ SKIP Next Q.10<br><br>Refused...77	Times _ _<br><br><br>Refused..77<br>N/A ……99	CHECK for unprotect-ed sex.<br><br>If Q7=3 AND Q8=Q9 SKIP to Q11<br><br>Else go to Q10	Both HIV positive....... 1<br>Not available….2<br>Too expensive …3<br>Partner objected.... ..4<br>Don't like them .... 5<br>Used other contraceptive … .... 6<br>Didn't think it was necessary... 7<br><br>Didn't think of it…………....8<br><br>Other (please specify)....... 9	Year……<br>Month…<br>….<br>Day……<br>…..	Year _ _<br>Month _ _<br>Day _ _<br><br>Refused.77	No……<br>2<br>Yes…...<br>1<br><br>Refused<br>.77	Positive.<br>.....1<br>Negative<br>….2<br>Don't know 88	No=2<br>Yes=1<br>I don't' know my status=99<br>Refused=77	No……………..<br>If No ⇨ SKIP to Section<br><br>Yes……………<br>If Yes ⇨ Repea questions for ne partner<br><br>Refused………<br>N/A …………..

								Refused…77						
NP2	Year _ _<br>Month _ _<br>Day _ _<br><br>Refused. 77<br>N/A … 99	Spouse ………..1<br>Cohabitating partner…… 2<br>Non-cohabitating regular partner…….3<br>Casual partner (friend/other).4<br>Commercial sex worker/client……..5<br>Other (specify)….6<br>(specify)<br>________<br><br>Refused. ……77<br>N/A……………99	Only once ….1<br>More than once but less than monthly.........2<br>About once per month………3<br>More than once per month but less than weekly……...4<br>Weekly……..5<br><br>Refused…77	Never …<br>…...1<br>Some of the time …<br>……..2<br>All of the time …<br>…..…3<br>Refused …<br>77	Times _ _<br><br>If 00⇨ SKIP Next Q.10<br><br>Refused...77	Times _ _<br><br><br>Refused..77<br>N/A ……99	CHECK for unprotect-ed sex.<br><br>If Q7=3 AND Q8=Q9 SKIP to Q11<br><br>Else go to Q10	Both HIV positive 1<br>Not available….2<br>Too expensive …3<br>Partner objected ..4<br>Don't like them 5<br>Used other contraceptive … 6<br>Didn't think it was necessary 7<br><br>Didn't think of it …<br>………...8<br><br>Other (please specify) 9<br><br>Refused…77	Year……<br>Month…<br>….<br>Day……<br>…..	Year _ _<br>Month _ _<br>Day _ _<br><br>Refused.77	No……<br>2<br>Yes…...<br>1<br><br>Refused .77	Positive.<br>.....1<br>Negative<br>….2<br>Don't know 88	No=2<br>Yes=1<br>I don't' know my status=99<br>Refused=77	No……………..2<br>If No ⇨ SKIP to Next Section<br><br>Yes…………….1<br>If Yes ⇨ Repeat questions for next partner<br><br>Refused………...7<br>N/A …………..…9
NP3	Year _ _<br>Month _ _<br>Day _ _<br><br>Refused. 77<br>N/A … 99	Spouse ………..1<br>Cohabitating partner…… 2<br>Non-cohabitating regular partner…….3<br>Casual partner (friend/other).4<br>Commercial sex worker/client……..5<br>Other (specify)….6<br>(specify)<br>________	Only once ….1<br>More than once but less than monthly.........2<br>About once per month………3<br>More than once per month but less than weekly……...4<br>Weekly……..5	Never …<br>…...1<br>Some of the time …<br>……..2<br>All of the time …<br>…..…3<br>Refused …<br>77	Times _ _<br><br>If 00⇨ SKIP Next Q.10<br><br>Refused...77	Times _ _<br><br><br>Refused..77<br>N/A ……99	CHECK for unprotect-ed sex.<br><br>If Q7=3 AND Q8=Q9 SKIP to Q11<br><br>Else go to Q10	Both HIV positive 1<br>Not available….2<br>Too expensive …3<br>Partner objected ..4<br>Don't like them 5<br>Used other contraceptive … 6<br>Didn't think it	Year……<br>Month…<br>….<br>Day……<br>…..	Year _ _<br>Month _ _<br>Day _ _<br><br>Refused.77	No……<br>2<br>Yes…...<br>1<br><br>Refused .77	Positive.<br>.....1<br>Negative<br>….2<br>Don't know 88	No=2<br>Yes=1<br>I don't' know my status=99<br>Refused=77	No……………..2<br>If No ⇨ SKIP to Next Section<br><br>Yes…………….1<br>If Yes ⇨ Repeat questions for next partner<br><br>Refused………...77<br>N/A …………..…99

		Refused. ……77<br>N/A……………99	Refused…77					was necessary<br>7<br><br>Didn't think of it<br>…<br>………...8<br><br>Other (please specify) 9<br><br>Refused…77						
NP4	Year _ _<br>Month _ _<br>Day _ _<br><br>Refused. 77<br>N/A … 99	Spouse ………..1<br>Cohabitating partner…… 2<br>Non-cohabitating regular partner…….3<br>Casual partner (friend/other).4<br>Commercial sex worker/client……...5<br>Other (specify)….6<br>*(specify)* ________<br><br>Refused.……77<br>N/A……………99	Only once ….1<br>More than once but less than monthly.........2<br>About once per month………3<br>More than once per month but less than weekly……...4<br>Weekly……..5<br><br>Refused…77	Never ……...1<br>Some of the time ………..2<br>All of the time ….. ……..…3<br>Refused …77	Times _ _<br><br>If 00⇨ SKIP Next Q.10<br><br>Refused...77	Times _ _<br><br><br>Refused..77<br>N/A ……99	CHECK for unprotect-ed sex.<br><br>If Q7=3 AND Q8=Q9 SKIP to Q11<br><br>Else go to Q10	Both HIV positive....... 1<br>Not available….2<br>Too expensive …3<br>Partner objected.... ..4<br>Don't like them .... 5<br>Used other contraceptive … .... 6<br>Didn't think it was necessary... 7<br><br>Didn't think of it…………...8<br><br>Other (please specify)....... 9<br><br>Refused…77	Year……<br>Month…<br>….<br>Day……<br>…..	Year _ _<br>Month _ _<br>Day _ _<br><br>Refused.77	No……<br>2<br>Yes…...<br>1<br><br>Refused .77	Positive. .....1<br>Negative ….2<br>Don't know 88	No=2<br>Yes=1<br>I don't' know my status=99<br>Refused=77	No……………..<br>If No ⇨ SKIP to Section<br><br>Yes……………<br>If Yes ⇨ Repea questions for ne partner<br><br>Refused………<br>N/A …………..

SR17. IN ADDITION TO THE PARTNERS YOU'VE DESCRIBED IN DETAIL ABOVE, HOW MANY ADDITIONAL PARTNERS IN THE PAST 6 MONTHS DID YOU HAVE?

NUMBER OF SEXUAL PARTNERS __

SR 18. SR. 18 IF YOU HAVE MENTIONED OTHER PARTNERS, HAVE YOU DISCLOSED YOUR HIV STATUS TO THEM?

[IF 0 SKIP TO SECTION 7, P1]

YES, FOR ALL OF THEM…………1
YES, FOR SOME OF THEM………..2
NO…………………………………...3
REFUSED……………………………77

N/A, I DON'T HAVE ANY OTHER SEXUAL PARTNERS…………99 [skip command would make this unnecessary]

"Please indicate how confident you are that you could perform each of the following tasks."

SECTION 7: SELF- EFFICACY FOR SAFE SEX, DISCLOSURE, AND PREVENTION ADVOCACY			
P 1	**HOW CONFIDENT ARE YOU THAT YOU** KNOW WHERE TO GET CONDOMS WHEREVER YOU ARE?	Not at all confident…………………………1<br>Not very confident .... 2<br>Somewhat confident .... 3<br>Very confident ……………………………... 4	
P 2	**HOW CONFIDENT ARE YOU THAT YOU** CAN MAKE SURE THAT YOU HAVE CONDOMS WITH YOU AT ALL TIMES?	Not at all confident.................................... 1<br>Not very confident .... 2<br>Somewhat confident .... 3<br>Very confident 4	
P 3	**HOW CONFIDENT ARE YOU THAT YOU** CAN USE CONDOMS CORRECTLY AND CONSISTENTLY EVEN WHEN YOU HAVE HAD A DRINK?	Not at all confident.................................... 1<br>Not very confident .... 2<br>Somewhat confident .... 3<br>Very confident 4	
P 4	**HOW CONFIDENT ARE YOU THAT YOU** CAN REFUSE TO HAVE SEX WITH YOUR PARTNER IF HE/SHE DOES NOT WANT TO USE CONDOMS	Not at all confident.................................... 1<br>Not very confident .... 2<br>Somewhat confident .... 3<br>Very confident... 4	
P 5	**HOW CONFIDENT ARE YOU THAT YOU** CAN USE CONDOMS WHEN YOU HAVE SEX WITH YOUR PARTNER(S)?	Not at all confindent.................................. 1<br>Not very confident .... 2<br>Somewhat confident .... 3<br>Very confident 4	
P 6	**HOW CONFIDENT ARE YOU THAT YOU** CAN TALK WITH YOUR PARTNER(S) ABOUT THE IMPORTANCE OF USING CONDOMS WHEN YOU HAVE SEX?	Not at all confindent.................................. 1<br>Not very confident .... 2<br>Somewhat confident .... 3<br>Very confident... 4	
P 7	**HOW CONFIDENT ARE YOU THAT YOU** CAN TALK WITH FAMILY AND/OR FRIENDS ABOUT THE IMPORTANCE OF USING CONDOMS CORRECTLY EVERYTIME THEY ARE HAVING SEX?	Not at all confident .... 1<br>Not very confident .... 2<br>Somewhat confident .... 3<br>Very confident 4	

P 8	**HOW CONFIDENT ARE YOU THAT YOU** CAN TALK WITH FAMILY AND/OR FRIENDS ABOUT THE IMPORTANCE OF GETTING TESTED FOR HIV?	Not at all confident.................................... 1<br>Not very confident .... 2<br>Somewhat confident .... 3<br>Very confident 4	
P 9	**HOW CONFIDENT ARE YOU THAT YOU** CANTALK WITH FAMILY AND/OR FRIENDS ABOUT SEEKING HIV CARE IF THEY HAVE HIV?	Not at all confident.................................... 1<br>Not very confident .... 2<br>Somewhat confident .... 3<br>Very confident.. 4	
P 10	**HOW CONFIDENT ARE YOU THAT YOU** CAN TALK WITH FAMILY AND/OR FRIENDS ABOUT THE IMPORTANCE OF DISCLOSING TO OTHERS ABOUT THEIR HIV STATUS?	Not at all confident…………………………..1<br>Not very confident .... 2<br>Somewhat confident .... 3<br>Very confident……………………………... 4	
P 11	**HOW CONFIDENT ARE YOU THAT YOU** CAN MAKE DECISIONS ABOUT WHEN IT IS THE RIGHT TIME TO TELL SOMEONE THAT YOU HAVE HIV?	Not at all confindent………………………..1<br>Not very confident .... 2<br>Somewhat confident .... 3<br>Very confident ……………………………4	
P 12	**HOW CONFIDENT ARE YOU THAT YOU** CAN MAKE GOOD DECISIONS ABOUT WHO TO TELL THAT YOU HAVE HIV?	Not at all confindent…………………………1<br>Not very confident .... 2<br>Somewhat confident .... 3<br>Very confident……………………………. 4	
P 13	**HOW CONFIDENT ARE YOU THAT YOU** CAN FOLLOW THROUGH WITH TELLING SOMEONE YOU HAVE HIV, ONCE YOU HAVE MADE THE DECISION THAT YOU WANT TO TELL THAT PERSON?	Not at all confindent………………………1<br>Not very confident .... 2<br>Somewhat confident .... 3<br>Very confident ……………………………... 4	

Have you been involved in an intimate relationship, such as married, living with someone, or dating within the past 6 months?

Yes………………………………………………………………..1
No………………………………………………………………2

If YES, go to Q. IPV1

If NO, skip to Section 9.

"Now, if you will permit me, I need to ask you some questions about your relationship with your partner/husband/wife. If we should come to a question that you do not want to answer, just let me know and we will go on to the next question."

SECTION 8: INTIMATE PARTNER VIOLENCE			
NIPV 1	HOW MUCH DO YOU APPRECIATE THE RELATIONSHIP WITH YOUR PARTNER?	Not at all .... 1<br>A little .... 2<br>Somewhat .... 3<br>Quite a lot .... 4<br>Very much .... 5<br>Refused to answer .... 77<br>N/A 99	
NIPV 2	DO YOU FEEL THAT YOU ARE APPRECIATED BY YOUR PARTNER?	Not at all .... 1<br>A little .... 2<br>Somewhat .... 3<br>Quite a lot .... 4<br>Very much .... 5<br>Refused to answer .... 77<br>N/A 99	
NIPV 3	DO YOU FEEL THAT YOUR PARTNER LISTENS TO YOU?	Not at all .... 1<br>A little .... 2<br>Somewhat .... 3<br>Quite a lot .... 4<br>Very much .... 5<br>Refused to answer .... 77<br>N/A 99	

NOW, I WILL ASK ABOUT DIFFERENT SITUATIONS THAT EXIST IN INTIMATE RELATIONSHIPS. PLEASE TELL ME IF ANY OF THE FOLLOWING HAS OCCURRED IN THE RELATIONSHIP YOU HAVE (OR HAVE HAD) WITH YOUR HUSBAND/WIFE OR INTIMATE PARTNER IN THE PAST SIX MONTHS..

A1) WAS JEALOUS OR ANGRY IF YOU TALK/ TALKED WITH …<br>(FEMALE RESPONDENT) OTHER MEN?<br>(MALE RESPONDENT) OTHER WOMEN?	*NIPV4 A*	NO 2<br>YES 1		A2) WERE YOU JEALOUS OR ANGRY IF YOUR PARTNER TALKED WITH …<br>(FEMALE RESPONDENT) .OTHER MEN?<br>(MALE RESPONDENT) OTHER WOMEN?	*NIPV4 B*	NO 2<br>YES 1	
B1) OFTEN SUSPICIOUS THAT YOU ARE UNFAITHFUL?	*NIPV5 A*	NO 2<br>YES 1		B2) WERE YOU OFTEN SUSPICIOUS THAT YOUR PARTNER WAS UNFAITHFUL?	*NIPV5 B*	NO 2<br>YES 1	
C1) YOUR PARTNER DID NOT ALLOW YOU TO MEET WITH FRIENDS?	*NIPV6 A*	NO 2<br>YES 1		C2) YOU DID NOT ALLOW YOUR PARTNER TO MEET WITH FRIENDS?	*NIPV6 B*	NO 2<br>YES 1	
D1) YOUR PARTNER PUT LIMITS TO YOUR COMMUNICATION WITH YOUR FAMILY?	*NIPV7 A*	NO 2<br>YES 1		D2) YOU PUT LIMITS TO YOUR PARTNERS COMMUNICATION WITH HIS/HER FAMILY?	*NIPV7 B*	NO 2<br>YES 1	
E1) INSISTED TO KNOW WHERE YOU ARE/YOU WERE EVERY MOMENT?	*NIPV8 A*	NO 2<br>YES 1		E2) YOU INSISTED TO KNOW WHERE YOUR PARTNER IS EVERY MOMENT?	*NIPV8 B*	NO 2<br>YES 1	
F1) DID NOT TRUST YOU WITH ANYTHING RELATED TO MONEY?	*NIPV9 A*	NO 2<br>YES 1		F2) YOUR DID YOU TRUST YOUR PARTNER WITH ANYTHING RELATED TO MONEY?	*NIPV9 B*	NO2<br>YES 1	

IF YOU WILL ALLOW ME, I WOULD LIKE TO ASK MORE QUESTIONS ABOUT YOUR RELATIONSHIP WITH YOUR HUSBAND/WIFE OR INTIMATE PARTNER (PERSON THAT USED TO BE YOUR HUSBAND/WIFE OR INTIMATE PARTNER). IF I ASK A QUESTION YOU DO NOT WISH TO RESPOND TO, PLEASE INFORM ME SO THAT I CAN ASK THE FOLLOWING QUESTION.

G1. HAS YOUR HUSBAND/WIFE OR INTIMATE PARTNER: IN THE PAST 6 MONTHS EVER:				G2 HAVE YOU IN THE PAST 6 MONTHS EVER:			
G1A) SAID OR DONE ANYTHING IN THE PRESENCE OF OTHER PEOPLE THAT EMBARRASSED OR SHAMED YOU?	*NIPV10 A*	NO 2<br>YES 1		G2A) SAID OR DONE ANYTHING IN THE PRESENCE OF OTHER PEOPLE THAT EMBARRASSED OR SHAMED YOUR HUSBAND/WIFE OR INTIMATE PARTNER?	*NIPV10 B*	NO 2<br>YES 1	
G1B) THREATENED TO HURT YOU/ OR SOMEONE THAT IS CLOSE TO YOU?	*NIPV11 A*	NO 2<br>YES 1		G2B) THREATENED TO HURT Y YOUR HUSBAND/WIFE OR INTIMATE PARTNER/OR SOMEONE CLOSE TO THEM?	*NIPV11 B*	NO 2<br>YES 1	
G1C) INSULTED YOU OR MADE YOU FEEL BAD ABOUT YOURSELF?	*NIPV12 A*	NO 2<br>YES 1		G2C) INSULTED YOU OR MADE YOUR HUSBAND/WIFE OR INTIMATE PARTNER FEEL BAD ABOUT THEMSELVES?	*NIPV12 B*	NO 2<br>YES 1	

H1. HAS YOUR HUSBAND/WIFE OR INTIMATE PARTNER EVER DONE ANY OF THE FOLLOWING IN THE PAST 6 MONTHS?				H2. HAVE YOU EVER DONE ANY OF THE FOLLOWING IN THE PAST 6 MONTHS TO YOUR HUSBAND/WIFE OR INTIMATE PARTNER			
H1A) PUSHED, SHOOK OR OR THREW SOMETHING AT YOU?	*NIPV13 A*	NO 2<br>YES 1		H2A) PUSHED, SHOOK OR OR THREW SOMETHING AT YOUR HUSBAND/WIFE OR INTIMATE PARTNER?	*NIPV13 B*	NO 2<br>YES 1	
H1B) SLAPPED YOU?	*NIPV14 A*	NO 2<br>YES 1		H2B) SLAPPED YOUR HUSBAND/WIFE OR INTIMATE PARTNER?	*NIPV14 B*	NO 2<br>YES 1	

H1C) TWISTED YOUR ARM OR PULLED YOUR HAIR?	*NIPV15A*	NO 2<br>YES 1		H2C) TWISTED ARM OR PULLED HAIR OR YOUR HUSBAND/WIFE OR INTIMATE PARTNER?	*NIPV15 B*	NO 2<br>YES 1	
H1D) HIT WITH FIST OR SOMETHING ELSE THAT COULD HURT YOU?	*NIPV16A*	NO 2<br>YES 1		H2D) HIT WITH FIST OR SOMETHING ELSE THAT COULD HURT YOUR HUSBAND/WIFE OR INTIMATE PARTNER?	*NIPV16 B*	NO 2<br>YES 1	
H1E) KICKED, DRAGGED ACROSS SURFACE OR BEAT YOU?	*NIPV17A*	NO 2<br>YES 1		H2E) KICKED, DRAGGED ACROSS SURFACE OR BEAT YOUR HUSBAND/WIFE OR INTIMATE PARTNER?	*NIPV17 B*	NO 2<br>YES 1	
H1F) TRIED TO CHOKE YOU OR BURN/SCALD YOU ON PURPOSE?	*NIPV18A*	NO 2<br>YES 1		H2F) TRIED TO CHOKE OR BURN/SCALD YOUR HUSBAND/WIFE OR INTIMATE PARTNER ON PURPOSE?	*NIPV18 B*	NO 2<br>YES 1	
H1G) THREATENED TO OR USED KNIFE, GUN OR ANY OTHER WEAPON TO HURT YOU?	*NIPV19A*	NO 2<br>YES 1		H2G) THREATENED TO OR USED KNIFE, GUN OR ANY OTHER WEAPON TO HURT YOUR HUSBAND/WIFE OR INTIMATE PARTNER?	*NIPV19 B*	NO 2<br>YES 1	
H1H) USED FORCE TO HAVE SEXUAL INTERCOURSE WITH YOU WHEN YOU DID NOT WANT TO?	*NIPV20A*	NO 2<br>YES 1		H2H) USED FORCE TO HAVE SEXUAL INTERCOURSE WITH YOUR HUSBAND/WIFE OR INTIMATE PARTNER WHEN THEY DID NOT WANT TO?	*NIPV20 B*	NO 2<br>YES 1	
H1I) FORCED YOU TO DO ANY ACT THAT WAS SEXUAL AND THAT YOU DID NOT WANT TO DO?	*NIPV21A*	NO 2<br>YES 1		H2I) FORCED YOUR HUSBAND/WIFE OR INTIMATE PARTNER TO DO ANY ACT THAT WAS SEXUAL AND THAT THEY DID NOT WANT TO DO?	*NIPV21 B*	NO 2<br>YES 1	

NOW I AM GOING TO ASK YOU SOME QUESTIONS ABOUT WHAT YOU THINK ABOUT YOUR AND OTHER PEOPLE'S ABILITIES TO CHANGE THE WAYS THEY DO THINGS

SECTION 9: STIGMA AND CHANGING BEHAVIOR			
CB 1	IF A PERSON LIVES WITH HIV HOW IMPORTANT DO YOU THINK THAT IT IS FOR HIM OR HER TO GET SUPPORT FROM THEIR FAMILY TO REDUCE STIGMA?	Very important .... 4<br>Quite important .... 3<br>Somewhat important................................. 2<br>A little important.. 1<br>Not at all important 0	
CB 2	IF A PERSON LIVES WITH HIV HOW IMPORTANT DO YOU THINK IT IS TO HIM OR HER TO GET SUPPORT FROM THEIR FRIENDS?	Very important .... 4<br>Quite important .... 3<br>Somewhat important................................. 2<br>A little important.. 1<br>Not at all important 0	
CB 3	HOW MUCH INFLUENCE DO YOU THINK THAT STIGMA HAS ON SPREADING THE VIRUS?	Very much .... 4<br>Quite a lot .... 3<br>Somewhat.. 2<br>A little .... 1<br>Not at all 0	
CB 4	HOW MUCH DO YOU THINK THAT YOU CAN INFLUENCE YOUR PARTNER'S BEHAVIOUR?	Very much .... 4<br>Quite a lot .... 3<br>Somewhat.. 2<br>A little .... 1<br>Not at all .... 0<br><br>N/A 99	

We are almost reaching the end of this interview. I will now ask you some questions about your use of alcohol in the past 6 months

12A	HAVE YOU USED ANY ALCOHOLIC DRINK IN THE PAST 6 MONTHS	Yes…………………………………………1<br>No…………………………………………..2	IF no end the interview here

SECTION 12: RAPID ALCOHOL PROBLEM SCREENING			
RAPS1	DURING THE LAST 6 MONTHS HAVE YOU HAD A FEELING OF GUILT OR REMORSE AFTER DRINKING?	Yes…………………………………………1<br>No…………………………………………..2	
RAPS 2	DURING THE LAST 6 MONTHS HAS A FRIEND OR FAMILY MEMBER EVER TOLD YOU ABOUT THINGS YOU SAID OR DID WHILE YOU WERE DRINKING THAT YOU COULD NOT REMEMBER?	Yes…………………………………………1<br>No…………………………………………..2	
RAPS 3	DURING THE LAST 6 MONTHS HAVE YOU FAILED TO DO WHAT WAS NORMALLY EXPECTED FROM YOU BECAUSE OF DRINKING?	Yes…………………………………………1<br>No…………………………………………..2	

RAPS 4	DO YOU SOMETIMES TAKE A DRINK WHEN YOU FIRST GET UP IN THE MORNING?	Yes………………………………………1<br>No………………………………………..2	
RAPS 5	DURING THE 6 MONTHS HAVE YOU LOST FRIENDS OR GIRLFRIENDS OR BOYFRIENDS BECAUSE OF DRINKING?	Yes………………………………………1<br>No………………………………………..2	

THIS IS THE END OF THE QUESTIONNAIRE. THANK YOU VERY MUCH FOR YOUR TIME AND YOUR WILLINGNESS TO ANSWER ALL THESE QUESTIONS. OVER THE NEXT TWO YEARS WHILE WE ARE DOING THE PROJECT, IT WOULD BE VERY HELPFUL TO FOLLOW THE DEVELOPMENTS IN DAR ES SALAAM BY INTERVIEWING YOU AGAIN AT SIX MONTHLY INTERVALS. WE APPRECIATE YOUR HELP.
